# Complex population evolutionary history of four cold-tolerant *Notopterygium* herb species in the Qinghai-Tibetan Plateau and adjacent areas

**DOI:** 10.1038/s41437-019-0186-2

**Published:** 2019-02-11

**Authors:** Mi-Li Liu, Yan-Ling He, Jordi López-Pujol, Yun Jia, Zhong-Hu Li

**Affiliations:** 1 Key Laboratory of Resource Biology and Biotechnology in Western China, Ministry of Education, College of Life Sciences, Northwest University, Xi’an, 710069 China; 2 Botanic Institute of Barcelona (IBB, CSIC-ICUB), Barcelona, 08038 Spain

**Keywords:** Genetic variation, Ecological genetics

## Abstract

Historical geological and climatic events are the most important drivers of population expansions/contractions, range shifts, and interspecific divergence in plants. However, the species divergence and spatiotemporal population dynamics of alpine cold-tolerant herbal plants in the high-altitude Qinghai-Tibetan Plateau (QTP) and adjacent areas remain poorly understood. In this study, we investigated population evolutionary history of four endangered *Notopterygium* herb species in the QTP and adjacent regions. We sequenced 10 nuclear loci, 2 mitochondrial DNA regions, and 4 chloroplast DNA regions in a total of 72 natural populations from the 4 species, and tested the hypothesis that the population history of these alpine herbs was markedly affected by the Miocene–Pliocene QTP uplifts and Quaternary climatic oscillations. We found that the four *Notopterygium* species had generally low levels of nucleotide variability within populations. Molecular dating and isolation-with-migration analyses suggested that *Notopterygium* species diverged ~1.74–7.82 million years ago and their differentiation was significantly associated with recent uplifts of the eastern margin of the QTP. In addition, ecological niche modeling and population history analysis showed that *N*. *incisum* and *N*. *franchetii* underwent considerable demographic expansions during the last glacial period of the Pleistocene, whereas a demographic contraction and a expansion occurred for *N. forrestii* and *N*. *oviforme* during the antepenultimate interglacial period and penultimate glacial period, respectively. These findings highlight the importance of geological and climatic changes during the Miocene–Pliocene and Pleistocene as causes of species divergence and changes in population structure within cold-tolerant herbs in the QTP biodiversity hotspot.

## Introduction

Historical and ecological factors such as geological and/or climatic processes are strongly linked with the origin and evolution of biodiversity (Coyne 1992; Excoffier et al. 2009; Mayr 1963; Slatkin and Excoffier 2012). The interactions among these processes can create geographic barriers but also new ecological niches, thereby providing opportunities for the origin and divergence of new species (Liu et al. 2013). Among these factors, mountain uplift and climatic oscillations have had important roles in shaping the current geographic distribution of biodiversity, especially in terms of speciation and population demography of plants (Hampe and Jump 2011; Hoffmann and Sgrò 2011). These events have frequently caused the fragmentation of species’ distributions and population isolation, thereby leading to decreased gene flow and the allopatric divergence of species (Coyne 1992; Rice and Hostert 1993). In addition, rapid climatic change events could have resulted in shifts in the effective population size of a species (e.g., Bai et al. 2018). In general, these evolutionary processes can be inferred by surveying the intraspecific genetic diversity and genealogies at multiple nuclear loci and organelle genome regions in the current geographic distributions of species (Excoffier et al. 2009; Slatkin and Excoffier 2012).

The Qinghai-Tibetan Plateau (QTP) sensu lato is a massive plateau with an area of about 2.5 million km^2^, stretching from the southern edge of the Himalayan Range to the northern edge of Kunlun Mountains, and from the eastern edge of Pamir and Karakoram Mountains to the eastern edge of Hengduan Mountains (Zhang et al. 2002). QTP is highly rich in species (including many endemics) due to its geological, climatic, and ecological diversity. Indeed, its southern and south-eastern sections harbor two of the Earth’s biodiversity hotspots, the Himalayas and Hengduan Mountains (Myers et al. 2000; Mittermeier et al. 2004). Therefore, this is an ideal area for studying the effects of different factors on species diversification and evolution. There is a growing consensus that the QTP began to uplift about 40–50 million years ago (Ma) due to the collision of the Indian subcontinent with the Eurasian plate (Favre et al. 2015; Mulch and Chamberlain 2006; Renner 2016; Yin and Harrison 2000); according to some recent viewpoints, the QTP could even have reached 4–5 km high since the mid-Eocene, about 40 Ma (Renner 2016). However, the eastern margin part of the QTP is geologically much newer, having started to uplift about after 10 Ma and continuing at least until late Pliocene (Favre et al. 2015; Sun et al. 2011; Xing and Ree 2017). Regardless of the exact timing, the complex history of the QTP uplift coupled with the formation of the monsoon climate have resulted in highly heterogeneous landforms and environmental gradients (Favre et al. 2015; Yin and Harrison 2000), with the formation of barriers and corridors for species exchange, as well as providing new ecological niches that stimulated the evolution of plant diversity (Xing and Ree 2017).

Some recent studies showed that the QTP uplift and the late Neogene complex climatic changes (e.g., the repeated glacial and interglacial cycles) have caused important population dynamics shifts and lineage divergence of alpine plant species that are distributed in the QTP itself and adjacent areas (Li et al. 2012; Liu et al. 2014; Ren et al. 2017). For instance, Zhao et al. (2016) suggested that the Himalayan-Tibetan Plateau uplift and subsequent climatic oscillations caused the allopatric divergence of two genera of alpine ginger, i.e., *Cautleya* Royle and *Roscoea* Smith. Phylogenetic and biogeographic analyses of *Spiraea* L. also indicated the close relationship between species diversification and the first two QTP uplift events (Khan et al. 2016). Moreover, some studies have emphasized the role of the QTP in the persistence of cold-tolerant plants, which survived in micro-refugia during glacial periods and expanded afterwards, shaping the current distribution patterns, e.g., *Aconitum gymnandrum* Maxim. (Wang et al. 2009a), *Potentilla glabra* G. Lodd. (Wang et al. 2009b), and *Primula tibetica* G. Watt (Ren et al. 2017). In addition, some studies of a variety of plant species in the QTP have shown that the areas with high genetic diversity and large numbers of haplotypes are located in the south-eastern refugial region of the plateau (i.e., the Hengduan Mountains) or other areas at the eastern edge of the plateau, whereas the genetic diversity is relatively low at the center of the plateau (Liu et al. 2014; Qiu et al. 2011), including *Pedicularis longiflora* Rudolph (Yang et al. 2008), *Juniperus przewalskii* Kom. (Zhang et al. 2005), *Picea crassifolia* Kom. (Meng et al. 2007), and *Paeonia delavayi* Franch./*P*. *ludlowii* (Stern and G. Taylor) D. Y. Hong (Zhang et al. 2018).

*Notopterygium* H. Boissieu (Umbelliferae) is an interesting model for studying species divergence and the spatiotemporal population dynamics of cold-tolerant herbal species in the high-altitude QTP and adjacent areas. In general, *Notopterygium* plants are insect-pollinated perennial herbs; their fruit type, a cremocarp with wings, favors dispersal by wind and water streams (Zhang 2013). In addition, the genus *Notopterygium* is endemic to China (synonymized as *Hansenia* Turcz.; see Pimenov et al. 2008) and it is mainly distributed in the QTP and adjacent alpine areas of Shaanxi, Gansu, and Sichuan. According to *Flora of China* (Wu and Raven 2005), this genus includes only six species: *Notopterygium* *incisum* C. C. Ting ex H. T. Chang, *Notopterygium* *franchetii* H. Boissieu, *Notopterygium* *oviforme* R. H. Shan, *Notopterygium* *forrestii* H. Wolff, *Notopterygium* *tenuifolium* M. L. Sheh and F. T. Pu, and *Notopterygium* *pinnatiinvolucellatum* F. T. Pu and Y. P. Wang. In particular, *N*. *incisum* and *N*. *franchetii* have similar distribution ranges within the QTP and adjacent mountain areas of western and central China. *N. incisum* grows in alpine forests or shrublands at high altitudes (3000–5000 m), whereas *N*. *franchetii* is found at much lower ones (1700–3500 m) on moist river banks and in mountain valleys. *N. oviforme* is distributed mainly in the Qingling mountain ranges in central and western China at elevations of 1800–2700 m. *N. forrestii* has a much smaller range, being only distributed in the southwestern part of Sichuan at altitudes of 2000–3000 m in forest margins and grasslands. The other two species, *N*. *tenuifolium* and *N*. *pinnatiinvolucellatum*, have very restricted distribution areas, with the former growing only in the valley meadows of Litang County in western Sichuan (at 4300 m) and the latter being distributed only in Xiaojin County in western Sichuan at about 3400 m (Wu and Raven 2005).

*Notopterygium* species are highly valued in China, because their roots and rhizomes are employed in traditional Chinese medicine (to dispel cold, expel wind, eliminate dampness, and relieve pain; Wang et al. 1995; Yang et al. 2006). However, in recent years, the high market demand for members of this genus together with the difficulty of their cultivation due to various life-history traits (habitat specificity, slow growth, and long life cycle) have led to significant population decline and/or range reduction (Zhou et al. 2010). Therefore, management and conservation measures are needed, which usually require knowledge of the levels and structure of the genetic diversity in plant populations as well as demographic information when possible (Falk and Holsinger 1991; Fenster and Dudash 1994; Lande 1988). In recent years, several studies of *Notopterygium* species based on molecular biology have been reported (Jia et al. 2017; Yang et al. 2017). Shahzad et al. (2017) determined the phylogeographic history and phylogenetic relationships of some *Notopterygium* species (those with sizable distribution areas) based on three chloroplast DNA (cpDNA) fragments and internal transcribed spacer (ITS) sequence variation. Phylogenetic analysis based on the ITS region showed that the four *Notopterygium* species (*N*. *incisum*, *N*. *oviforme*, *N*. *franchetii*, and *N*. *forrestii*) constituted four independently evolved genetic lineages that corresponded to four taxonomically recognized species. In contrast, the phylogenetic analysis based on cpDNA was not able to separate *N*. *oviforme* and *N*. *franchetii* (Shahzad et al. 2017), probably due to the low resolutions of molecular markers used. In addition, these previous studies did not focus on aspects such as speciation history, interspecific gene flow, and deep evolutionary history in *Notopterygium* species.

In this study, we investigated the same four *Notopterygium* species (*N*. *incisum*, *N*. *oviforme*, *N*. *franchetii*, and *N*. *forrestii*) across their entire geographic distributions in the high-altitude QTP and adjacent areas. We employed maternally inherited cpDNA sequences, mitochondrial DNA (mtDNA) sequences, and biparentally inherited nuclear genes, to determine the population genetic variability, evolutionary history, and species divergence in these alpine medicinal herbal plants. We mainly addressed the following questions: (1) how did the effects of the QTP uplift contribute to genetic variation and species divergence among *Notopterygium* populations? (2) how did Quaternary climatic changes (including those of ongoing global warming) affect the population demography and the distribution range of these four *Notopterygium* species? and (3) how did the interspecific gene flow proceed (and how was this related to the speciation processes) in the cold-tolerant *Notopterygium* herb species?

## Materials and methods

### Population sampling and molecular data

Initially, 340 individuals were collected from 72 wild populations (26 populations of *N*. *incisum*, 27 populations of *N*. *franchetii*, 15 populations of *N*. *oviforme*, and 4 populations of *N*. *forrestii*; the details of the sampled populations are shown in Table S1) throughout their distribution ranges (some populations and individuals come from the same materials of Shahzad et al. 2017). All sampled populations were separated by at least 50 km from each other and individuals from each population were spaced at least 50 m apart. The material collected comprised fresh leaves, which were dried rapidly in silica gel and stored until DNA isolation. The vouchers of plant materials were deposited into the herbarium of Northwest University (China) (Table S1). Total genomic DNA was isolated from the leaf tissues using a plant genomic DNA extraction kit (Tiangen, Beijing, China) or the modified CTAB protocol (Doyle and Doyle 1990). All individuals were amplified and sequenced at one cpDNA fragment (OG28079), two mtDNA fragments (OG917 and OG537), and ten single copy nuclear loci (35, 9122, 25629, 25679, 29206, 32125, 51964, OG29101, OG29960, and OG29988). These nuclear loci were selected from the comparative transcriptome analysis between two *Notopterygium* species, *N*. *incisum* and *N*. *franchetii* (Jia et al. 2017). The single-copy orthologous genes between the two species were determined by OrthoMCL analysis (Li et al. 2003) (Table S2). PCR amplification was performed in a 20 μL system comprising 11 μL of 2 × *Taq* PCR MasterMix, 0.3 μM of each primer, 10–50 ng template DNA, and 7.4 μL ddH_2_O. All amplifications were conducted in a PTC-2000 thermal cycler (MJ Research) as follows: 5 min at 94 °C, followed by 32 cycles at 94 °C for 40 s, 40 s at the specific annealing temperature (Tm) for each marker, and 90 s at 72 °C, with a final extension at 72 °C for 10 min. The PCR products were then purified and sequenced using an ABI 3730xl genetic analyzer (Tsingke Biological Technology, Xi’an, China). We sequenced the PCR products directly on both strands for the cpDNA and mtDNA fragments. The PCR products were cloned into *pGEM* T-easy vectors (Promega) for the nuclear genes where heterozygous individuals existed. We randomly selected five clones for Sanger sequencing. The newly obtained sequences were submitted to GenBank under accession numbers MK312210–MK312239, MK305312–MK305813, and MK258173–MK258185. Meanwhile, we have also combined the three previously published (Shahzad et al. 2017) cpDNA sequences (*trnS-trnG*, *matK*, and *rbcL*) with the currently obtained cpDNA OG28079 (*ndhF*) for further population genetic analysis of *Notopterygium* species.

### Genetic diversity and neutrality tests

All sequences were checked and aligned with BioEdit v7.0.9.0 (Hall 1999) and all nuclear gene sequences were assigned to coding and noncoding regions by aligning the genomic sequences against their corresponding mRNAs. Polymorphic sites of nuclear loci were further phased to verify the results of cloning sequencing, which were employed by the Bayesian statistical method PHASE with 1000 Markov chain Monte Carlo (MCMC) and 1000 burn-in iterations in DnaSP v5.10 (Librado and Rozas 2009). We also used DnaSP to estimate the basic population genetic parameters, such as the number of segregating sites (*S*), Watterson’s parameter (*θ*_w_; Watterson 1975), nucleotide diversity (*π*; Tajima 1983), and the minimum number of recombinant events (*R*_m_; Hudson and Kaplan 1985). Genetic diversity based on nuclear genes was investigated by estimating the haplotype ( = allele) number (*N*_h_) and diversity (*H*_d_) based on the number of segregating sites (Depaulis and Veuille 1998; Depaulis et al. 2001; Fu 1997).

In addition, we tested the neutral evolution patterns for nuclear loci (but also for cpDNA and mtDNA) using various statistics in DnaSP, including Tajima’s *D* statistic (Tajima 1989), Fu and Li’s D* and F* (Fu and Li 1993), and Fay and Wu’s *H* (Fay and Wu 2000). According to previously published phylogenetic results, homologous sequences from *Daucus carota* L. subsp. *sativus* (Hoffm.) Arcang. (LOC108202681, LOC108225228, LOC108197207, LOC108227844, LOC108193745, LOC108227632, and LOC108210968), *Heracleum moellendorffii* Hance (LOC108225228), *Pleurospermum franchetianum* Hemsl. (LOC108995448), and *Pleurospermum prattii* H. Wolff (LOC108224998) were used as outgroups (Iorizzo et al. 2013; Xue et al. 2007; Yang et al. 2017). We examined the likelihood of natural selection acting on ten nuclear loci at the species level using the recently developed maximum frequency of derived mutations (MFDM) test; this test is not affected by the confounding impacts of bottlenecks and size expansions (Li 2011).

### Population structure and phylogenetic analyses

The genetic structure of the four *Notopterygium* species was investigated to assess the correspondence between genotypic clustering and taxonomic delimitation based on variations in nuclear loci using STRUCTURE v2.3 (Hubisz et al. 2009). This program employs a Bayesian algorithm to infer the true number of clusters (*K*) in a sample of individuals. *K*-values were explored from 1 to 10 using 20 independent runs per *K*, employing an admixture model with a burn-in of 100,000 iterations and a run length of 200,000 iterations. The best *K*-value was estimated using the Δ*K* method (Evanno et al. 2005) and by choosing the smallest *K* after the log probability of the data values [ln Pr(*X*|*K*)] reached a plateau (Pritchard et al. 2010). The Clumpp v1.1.2 program (Jakobsson and Rosenberg 2007) was used to combine the results from the 20 repetitions of the best *K*. The Distruct v1.1 program (Rosenberg 2004) was used to graphically display the results produced by Clumpp.

In order to quantify the extent of differentiation among species, we first estimated Wright’s fixation index *F*_ST_ (Wright 1951) and the net sequence divergence (*D*a; Nei 1987). *F*-statistics were computed for each gene based on the haplotype ( = allele) frequencies as variance component ratios using the locus-by-locus analysis of molecular variance (AMOVA; Excoffier et al. 1992) approach implemented in Arlequin v3.1 (Excoffier et al. 2005). The net sequence divergence (*D*a) was estimated using DnaSP.

Haplotypes of the cpDNA and mtDNA, and nuclear genes were identified with DnaSP v5.10 and median-joining networks of sequences were constructed using Network v5.0 (Bandelt et al. 1999). We used ArcGIS v10.2 (ESRI, Redlands, CA, USA) to visualize the haplotype results superimposed on maps. Hierarchical analyses of genetic differentiation were investigated for cpDNA, mtDNA, and nuclear loci based on AMOVA, which was performed in Arlequin v3.1 using pairwise *F*_ST_ as the distance measure with 10,000 permutations, to investigate the genetic variation among and within populations for each species (Excoffier and Lischer 2010). We estimated the average mtDNA and cpDNA gene diversity within populations (*H*_S_), total gene diversity (*H*_T_), and the values of genetic differentiation over all populations (*G*_ST_), as well as the values of differentiation by considering genetic distance (*N*_ST_) using PERMUT (Pons and Petit 1996). The significance for comparison test between *G*_ST_ and *N*_ST_ was assessed by a nonparametric permutation procedure with 1000 permutation tests.

The phylogenetic relationships among species were inferred based on separate nuclear haplotypes ( = alleles) using a Bayesian inference method. The best-fit model of nucleotide substitution was determined by jModelTest v2.1.5 based on Akaike’s information criterion and the MrBayes v3.2 program (Ronquist et al. 2012) was then used for the Bayesian inference. MCMC analyses were performed with four chains each for 10,000,000 generations, with sampling every 1000 generations and a burn-in of 25%. The results were visualized using FigTree v1.3.1 (Rambaut 2016). The program RAxML v7.2.8 (Stamatakis 2006) was also used to determine the phylogenetic relationships among the four *Notopterygium* species under the maximum-likelihood approach using the concatenated nuclear genes. The rapid bootstrapping and the best ML tree search were conducted with 100,000 of randomized maximum parsimony starting trees and 1000 bootstrap replicate tests.

In addition, the divergence time estimation was performed in BEAST v1.7.5 (Drummond and Rambaut 2007) using two datasets, the combined four chloroplast fragments, and the concatenated ten nuclear loci. The BEAST analyses were conducted under the uncorrelated log-normal relaxed clock approach with a Yule tree prior and appropriate nucleotide substitution model (GTR + G + I). Two independent replications each with 50,000,000 generations were run with sampling every 1000 generations and the first 25% were discarded. We used the two fossil-based calibration points for temporally normal constraints: the split between *Steganotaenia* Hochst. and *Bupleurum* L. at 55.8 Ma (Gruas-Cavagnetto and Cerceau-Larrival 1984) and the split between Umbelliferae and Araliaceae at 69 Ma (Yi and Kim 2012). Meanwhile, due to the lack of nuclear data for the outgroups used in chloroplast markers analysis, we used an evolutionary rate (*μ* = 6.1 × 10^−9^) instead of calibration points to calculate the divergence time (*Asteraceae*; Sang et al. 1994,) based on nuclear data.

### Demographic history

To determine the divergence history of *Notopterygium* species, we used IMa2 software based on the isolation-with-migration (IM) model (Hey and Nielsen 2004; Hey 2010; Nielsen and Wakeley 2001; Wakeley and Hey 1997). IMa2 implements a coalescent-based method that employs MCMC sampling of gene genealogies to estimate the population parameters scaled by the mutation rate per locus per generation (*μ*), including the contemporary and ancestral effective population sizes [*θ*, where *θ* = 4*N*_e_*μ* (for nuclear genes) and *N*_e_ is the effective population size], migration rates (*m* = *M*/*μ*, where *M* is the effective migration rate per generation), and divergence times (*t* = *Tμ*, where *T* is the divergence time in years before present) among populations. We analyzed the species in a pairwise manner using a basic two-population model and, because of the absence of fossils or any published mutation rate of Umbelliferae, we used an evolutionary rate of *μ* = 6.1 × 10^−9^ per site per generation, to calculate the divergence times (*Asteraceae*; Sang et al. 1994). The average generation time was set to three years according to previous experimental investigations of *Notopterygium* (Shahzad et al. 2017).

**Fig. 1:**
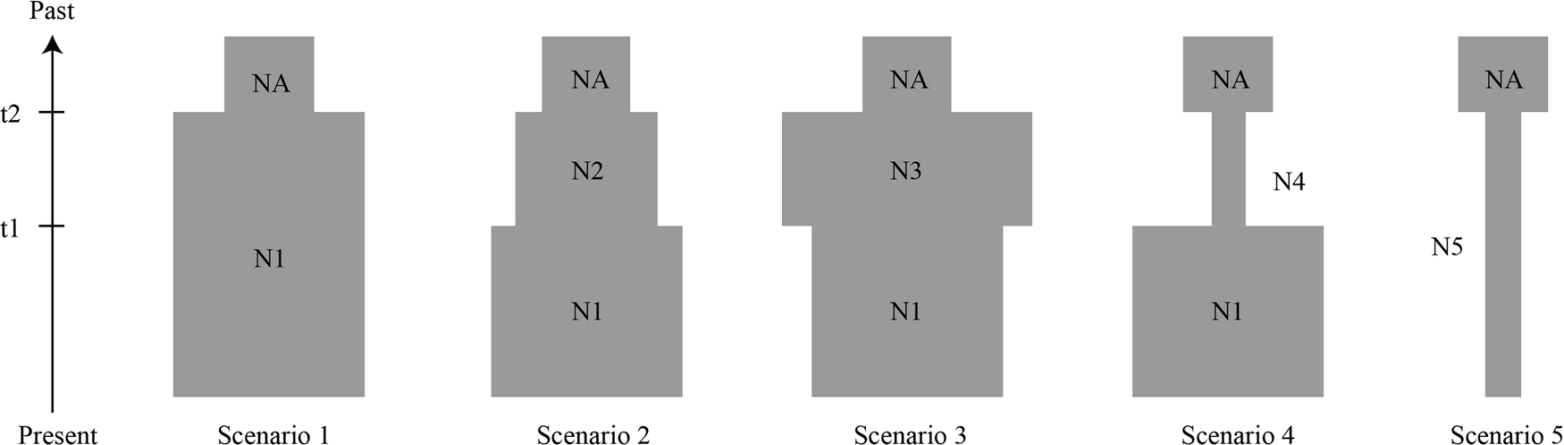
Tested historical demographic models proposed for the populations of the four studied *Notopterygium* species

In addition, to investigate the early demographic histories of these four species, we designed and examined five plausible scenarios of demographic changes using approximate Bayesian computation (ABC) in DIYABC v1.0 (Cornuet et al. 2010), based on the sequence data obtained for the ten nuclear loci. These five scenarios are based on the results of the related demographic analyses; for instance, Isolation-with-Migration (IMa2) analysis showed that the four species had undergone observable population expansions and a low level of nucleotide variability, and neutral tests showed that the four *Notopterygium* species might have experienced relatively ancient bottlenecks. All five scenarios were simulated under the framework of a single population by assuming the same initial population size (NA). Scenario 1 assumed ancient population growth (t2), followed by a larger and stable population size (N1). Scenario 2 assumed an ancient population growth (t2) followed by a larger but stable population size (N2), and finally a recent expansion (t1, N1). Scenario 3 assumed an ancient population growth (t2) and a subsequent larger but stable population size (N3), followed by a recent bottleneck (t1, N1). Scenario 4 assumed an ancient bottleneck event (t2) with a subsequent smaller stable population size (N4) and a recent expansion (t1, N1). Finally, scenario 5 assumed an ancient bottleneck event (t2) and a smaller but stable population size (N5) (Fig. 1). The prior distributions of the demographic parameters are listed in Table S3.

### Ecological niche modeling

We used MaxEnt v3.3.3k (Phillips et al. 2006; Phillips and Dudík 2008) to predict the current, Last Glacial Maximum (LGM, 0.021–0.018 Ma), Last Interglacial (LIG, 0.140–0.120 Ma), and future (year 2050) potential distributions of *Notopterygium* species. For the LGM period, we used paleoclimatic layers simulated by the Community Climate System Model Version Version 4 (CCSM4; Gent et al. 2011), the Model for Interdisciplinary Research on Climate Earth System Model (MIROC-ESM; Watanabe et al. 2011), and the New Earth System Model of the Max Planck Institute for Meteorology (MPI-ESM-P: http://www.mpimet.mpg.de/en/science/models/mpi-esm/). For the LIG period, we used the dataset of Otto-Bliesner et al. (2006). To predict potential shifts of the geographic distribution that might be caused by global climate change by the year 2050, we used three models: CCSM4, the NOAA Geophysical Fluid Dynamics Laboratory Coupled Model 3 (Donner et al. 2011), and MPI-ESM-P. The three models were run in the most extreme representative concentration pathways (RCPs), RCP 2.6 and RCP 8.5 (Collins et al. 2013). Layers for 19 bioclimatic variables (Table S4) of these models plus for the current time (1960–1990) were downloaded at 2.5 arc-min resolution (or later transformed to this resolution, such as for the LIG model) from the WorldClim website (www.worldclim.org; Hijimans et al. 2005) for the study area (12–56°N and 71–139°E).

The sites of occurrence for *Notopterygium* species were collected from the Chinese Virtual Herbarium (http://www.cvh.ac.cn/) and our sampling location information (for detailed information, see Supplementary Table S5). We removed duplicate records from the same locality to reduce the effects of spatial autocorrelation. A total of 109 sampling sites of *N. incisum*, 45 sites of *N. franchetii*, and 15 localities of *N. oviforme* were used for the ecological niche modeling (ENM). We did not model the distribution of *N*. *forrestii*, because the number of localities (four) was not sufficient to make reliable predictions. To avoid multicollinearity, we assessed Pearson’s correlation coefficients between all of the layers within the study area; the selection of variables from pairs or groups of highly correlated ones (*r* ≥ |0.9|) was done on the basis of their relative contribution to the model (percent contribution, jackknife tests of variable importance) and the shape of their response curves, making sure that the top most influential variables for each species were selected. Variables with neither significant contribution to the models (with values of percent contribution below 5) nor clear response (i.e., with response curve flat or nearly flat) were not considered. The final selected variables that were used to detect changes in the distribution ranges of *N*. *incisum*, *N*. *franchetii*, and *N*. *oviforme* were bio1, bio2, bio3, bio4, bio5, bio12, bio14, bio15, and bio18.

We used the default parameters for MaxEnt and employed the “subsample” method (setting the number of replicates to 20), with 75% of the species records for training and 25% for testing the model. The overall model performance was assessed using the area under the curve (AUC) of the receiver operating characteristic. AUC scores range between 0.5 (randomness) and 1 (exact match), and a value above 0.9 is considered a good performance of the model (Swets 1988). As threshold rule, we chose applying the maximum sensitivity plus specificity logistic threshold, which is very robust with all types of data (Liu et al. 2016). All ENM predictions were visualized in ArcGIS.

### Climatic niche comparisons

A niche comparison analysis was performed to test whether the selection of different types of climatic niches may have contributed to divergence of *Notopterygium* species. The analysis was based on environmental space (E-space) under the PCA-env approach developed by Broennimann et al. (2012). To conduct the analysis, previous R scripts reported in Broennimann et al. (2012) and Silva et al. (2016) with slight modifications were followed.

As input data, we used the same as for the ENM assessment: the nine relatively uncorrelated climatic variables (see above) and the geographical coordinates for the three *Notopterygium* species that have more than ten occurrence records. The climatic background areas were defined as minimum convex polygons with a buffer size of 0.3°, such as proposed in Silva et al. (2016). The PCA-env was constructed on an environmental space of 500 × 500 grid-cell resolution, in which the three realized niches were simultaneously represented. The niche overlap values observed in the PCA-env by pairs of species were calculated using the Schoener’s *D*_S_; this metric may range from 0 to 1, representing no overlap and equal niches, respectively (Schoener 1970; Warren et al. 2008). In addition, statistical tests of niche equivalency (considering only the occurrences) and similarity (considering both occurrences and background climates) were also computed. These tests compare observed (*D*_obs_) with 100 randomly simulated overlap values (*D*_sim_) under a null distribution. When *D*_obs_ is greater or smaller than *D*_sim_ (with a *P* < 0.05), niche conservatism or divergence could be proposed, respectively. Alternatively, when *D*_obs_ is within 95% of *D*_sim_ (*P* > 0.05), the hypothesis of conservatism or divergence can be neither accepted nor rejected with confidence. Finally, we quantified the three niche dynamic metrics of niche unfilling, stability, and expansion.

## Results

### Genetic diversity and neutrality tests

Four cpDNA, 2 mtDNA, and 10 nuclear loci were finally analyzed from 340 individuals, which represented 72 natural populations of four *Notopterygium* species. The concatenated fragments of cpDNA and mtDNA comprised 1804 bp and 755 bp, respectively. The sequenced nuclear loci ranged from 214 to 842 bp, with a total concatenated length of 4580 bp after excluding gaps and missing data. In general, the levels of nucleotide polymorphism (*θ*_wt_ and *π*_t_) over all nuclear loci were higher in *N*. *incisum* (0.0065 and 0.0031, respectively), followed by *N*. *franchetii* (0.0048 and 0.0027) and *N*. *oviforme* (0.0043 and 0.0035), and finally those of *N*. *forrestii* (0.0021 and 0.0023) (Table 1). The diversity at silent sites (*π*_s_) was ~3 times greater than that in nonsynonymous sites (*π*_n_), where each gene had a *π*_n_⁄*π*_s_ ratio of < 1 in all four species (Table 1). The nucleotide polymorphism patterns (*θ*_wt_ and *π*_t_) were similar over the cpDNA and mtDNA loci (Table 2), with the four *Notopterygium* species generally showing low levels of nucleotide variability within populations (Table S6).

**Table 1:** Nucleotide variation for *N. incisum*, *N. franchetii*, *N. oviforme*, and *N. forrestii*

Species	Total					Nonsynonymous sites		Silent sites		*R*_m_
	*N*	*L*	*S* (Singl.)	*θ*_wt_ (SD)	*π*_t_ (SD)	*θ*_wn_	*π*_n_	*θ*_*s*_	*π*_*s*_	
*N. incisum*	162	448	16 (2.7)	0.0065 (0.0021)	0.0031 (0.0003)	0.0026	0.0024	0.0072	0.0036	1.9
*N. franchetii*	163	448	10.3 (1.2)	0.0048 (0.0016)	0.0027 (0.0004)	0.0013	0.0015	0.0066	0.0050	1.9
*N. oviforme*	69	458	9.8 (1.1)	0.0043 (0.0018)	0.0035 (0.0004)	0.0027	0.0016	0.0065	0.0055	1.4
*N. forrestii*	20	454	3.6 (0.3)	0.0021 (0.0011)	0.0023 (0.0004)	0.0011	0.0012	0.0029	0.0032	0.6

Nucleotide variation for *N. incisum*, *N. franchetii*, *N. oviforme*, and *N. forrestii*, where the number of samples (*N*), length (*L*), number of segregating sites (*S*), and the minimum number of recombination events (*R*_m_) are averaged over all nuclear loci. Watterson’s *θ* (*θ*_w_) and the average number of pairwise nucleotide differences (*π*) are per site estimates of the population scaled mutation parameter *θ* averaged over all nuclear loci

**Table 2:** Nucleotide variation for *N. incisum*, *N. franchetii*, *N. oviforme*, and *N. forrestii*

cpDNA			Diversity sites					Neutrality test		
Species	*N*	*L*	*Θ*_wt_ (SD)	*π*_t_ (SD)	*N*_h_	*H*_d_	*D*	*H*	D*	F*
*N*. *incisum*	86	1761	0.00158 (0.00057)	0.00119 (0.00007)	22	0.8569	− 0.68752	− 0.44444	− 1.75001	− 1.63040
*N*. *franchetii*	90	1776	0.00100 (0.00040)	0.00044 (0.00100)	16	0.7803	− 1.41216	− 0.01131	0.58640	− 0.11769
*N. oviforme*	39	1778	0.00213 (0.00080)	0.00171 (0.00025)	17	0.9528	− 0.63165	− 0.02001	0.36015	0.04021
*N. forrestii*	10	1778	0.00040 (0.00030)	0.00040 (0.00018)	3	0.5111				
mtDNA			Diversity sites					Neutrality test		
Species	*N*	*L*	*Θ*_wt_ (SD)	*π*_t_ (SD)	*N*_h_	*H*_d_	*D*	*H*	D*	F*
*N. incisum*	98	755	0.00103 (0.00056)	0.00070 (0.00009)	4	0.4841	− 0.64264	0.26678	− 2.66557	− 2.36971
*N. franchetii*	101	755	0.00026 (0.00026)	0.00047 (0.00006)	2	0.3552	0.96441	0.2505	0.49171	0.73856
*N. oviforme*	56	755	0.00029 (0.00029)	0.00066 (0.00003)	2	0.4987	1.68499	-0.16623	0.53364	1.00474
*N. forrestii*	8	755	0	0	1	0				

Nucleotide variation for *N. incisum*, *N. franchetii*, *N. oviforme*, and *N. forrestii* over cpDNA and mtDNA loci. *D* Tajima’s *D*, D* Fu and Li’s D*, F* Fu and Li’s F*, *H* Fay and Wu’s *H*, *H*_d_ haplotype diversity, *L* length, *N* number of samples, *N*_h_ number of haplotypes, *θ*_wt_ Watterson’s *θ*, *π*_t_ total nucleotide diversity

The estimates of average within-population cpDNA diversity (*H*_S_) and total cpDNA diversity (*H*_T_) in *N*. *incisum*, *N*. *oviforme*, and *N*. *franchetii* were 0.227 and 0.801, 0.205 and 0.910, and 0.177 and 0.371, respectively. In terms of within-population and total mtDNA diversity, *N*. *incisum* (*H*_S_ = 0.142) and *N*. *oviforme* (*H*_T_ = 0.529) had the highest values among the taxa studied (Table 3), respectively. Significant phylogeographic structure was detected in *N*. *incisum* and *N*. *franchetii* (i.e., *N*_ST_ > *G*_ST_, *P* < 0.05; Table 3).

**Table 3:** Estimates of average genetic diversity

Regions	*H*_S_	*H*_T_	*G*_ST_	*N*_ST_	*P*-value
**cpDNA**					
Four species	0.184 (0.0389)	0.877 (0.0265)	0.790 (0.0437)	0.959 (0.0094)^**^	—
*N. incisum*	0.227 (0.0743)	0.801 (0.0357)	0.717 (0.0918)	0.821 (0.0627)^*^	0.025
*N. franchetii*	0.177 (0.0610)	0.371 (0.0989)	0.522 (0.1569)	0.685 (0.1359)^*^	0.017
*N. oviforme*	0.205 (0.0767)	0.910 (0.0631)	0.774 (0.0761)	0.817 (0.0772)	0.301
*N. forrestii*	—	—	—	—	—
**mtDNA**					
Four species	0.063 (0.0226)	0.427 (0.0596)	0.852 (0.0526)	0.836 (0.0627)	—
*N. incisum*	0.142 (0.0555)	0.452 (0.0694)	0.685 (0.1171)	0.634 (0.1322)	0.652
*N. franchetii*	0.012 (0.0123)	0.271 (0.0960)	0.954 (0.0484)	0.954 (0.0484)	—
*N. oviforme*	0.033 (0.0333)	0.529 (0.0259)	0.937 (0.0630)	0.937 (0.0630)	—
*N. forrestii*	—	—	—	—	—

Estimates of average genetic diversity within populations (*H*_S_), total genetic diversity (*H*_T_), interpopulation differentiation (*G*_ST_), and the number of substitution types (*N*_ST_) (mean ± SE in parentheses) for cpDNA and mtDNA

*cpDNA* chloroplast DNA, *mtDNA* mitochondrial DNA. ^**^P < 0.001 and ^*^P < 0.05

The values of Tajima’s *D*, and Fay and Wu’s *H* were negative at most nuclear loci (although most of the values were not significant; Table 4). The negative average values for *N. incisum*, *N. franchetii*, and *N. oviforme* indicated that variants were skewed toward both low-frequency values (negative *D*) and high-frequency-derived values (negative *H*). In addition, the average MFDM test values were not significant for all nuclear loci, which suggested no likelihood of natural selection acting on individual loci at the species level.

**Table 4:** Haplotype ( = allele) diversity and neutrality tests for *N. incisum*, *N. franchetii*, *N. oviforme*, and *N. forrestii*

Species	Haplotype diversity		Neutrality tests				
	*N*_h_	*H*_d_	*D*	*H*	D*	F*	MFDM
*N. incisum*	19	0.5686	− 1.4157	− 5.6354	− 0.0636	− 0.6896	0.1394
*N. franchetii*	15	0.5020	− 0.9291	− 3.8564	0.2614	− 0.2151	0.2333
*N. oviforme*	11	0.5520	− 0.6497	− 2.7430	0.2274	− 0.0790	0.2566
*N. forrestii*	5	0.4800	0.1478	− 0.2488	0.5559	0.5095	—

Haplotype ( = allele) diversity and neutrality tests for *N. incisum*, *N. franchetii*, *N. oviforme*, and *N. forrestii*, where the number of haplotypes (*N*_h_), haplotype diversity (*H*_d_), Tajima’s *D* (*D*), Fu and Li’s D* (D*), Fu and Li’s F* (F*), Fay and Wu’s *H* (*H*), and MFDM are averaged across all nuclear loci

### Genetic differentiation and population structure

**Fig. 2:**
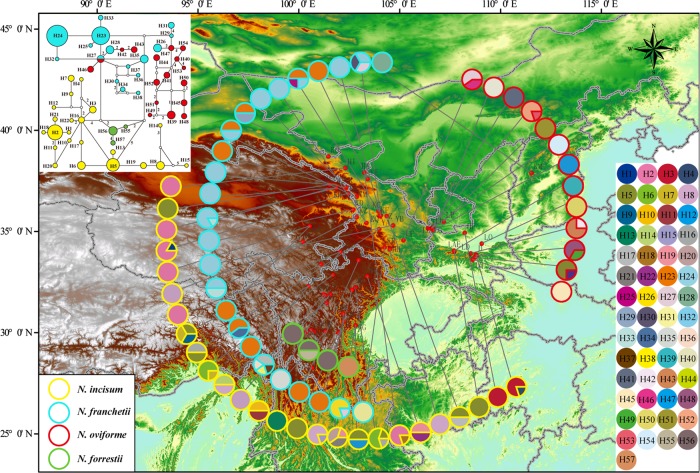
Geographic distributions of the cpDNA haplotypes for the four studied *Notopterygium* species

**Fig. 3:**
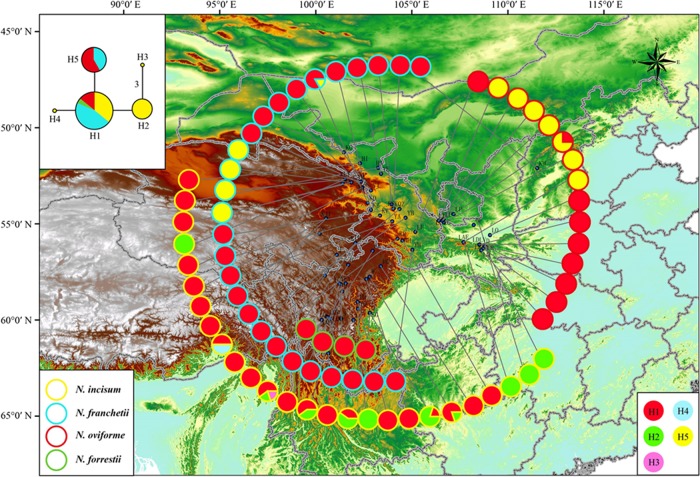
Geographic distributions of mtDNA haplotypes for the four studied *Notopterygium* species

Up to 57 cpDNA sequence haplotypes were identified among all studied individuals from the four *Notopterygium* species (Fig. 2). *N. incisum* (H1–H22), *N*. *franchetii* (H23–H38), *N*. *oviforme* (H27 and H39–H54), and *N*. *forrestii* (H55–H57) had 22, 15, 16, and 3 private haplotypes, respectively, whereas one haplotype (H27) was shared between *N*. *franchetii* and *N*. *oviforme*. In total, five haplotypes were identified based on the mtDNA (Fig. 3). Mitotype H1 had a central position in the network and it was shared widely by all four *Notopterygium* species, whereas mitotype H5 was shared by *N*. *franchetii* and *N*. *oviforme*, and only *N*. *incisum* had private mitotypes (H2–H4). Moreover, AMOVA based on the cpDNA data showed that the genetic variation occurred mainly among species (63.8%), whereas most of the variation in terms of mtDNA was due to differences among populations within species (54.6%; Table S7).

The levels of nuclear genetic divergence between species also varied across loci (Table S8), in agreement with the great differences between the haplotype ( = allele) networks that were obtained for each locus, both in terms of number of haplotypes and network structure (Fig. S1). Indeed, 8–98 haplotypes per locus were identified among all of the samples from the 4 species (Fig. S1). At most loci, *N*. *incisum* and *N*. *forrestii* had the highest number of private haplotypes, whereas *N*. *franchetii* and *N*. *oviforme* haplotypes were mostly shared. However, AMOVA showed that at all nuclear loci, most of the variability was explained by genetic differences between the four species (63.6–94.2%) (*P* < 0.001, Table S7).

**Fig. 4:**
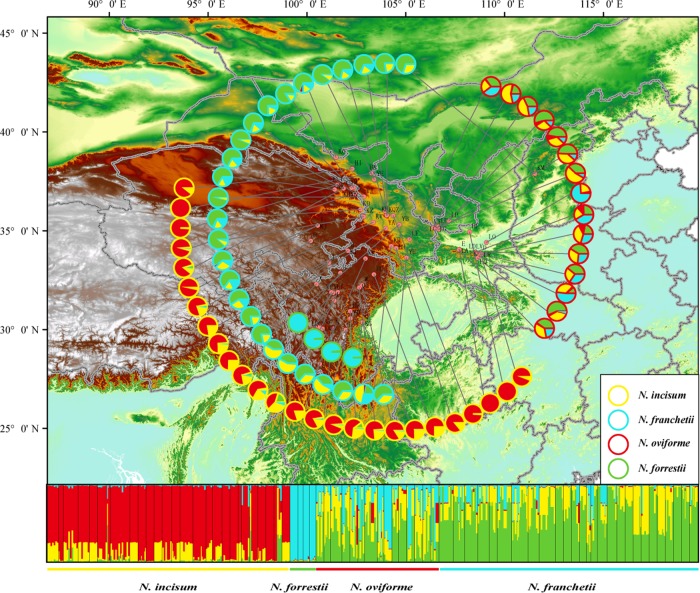
Geographic distributions of the four genetic clusters (*K* *=* 4) identified with the program STRUCTURE in the four studied *Notopterygium* species

The population structure of the four species was investigated further with STRUCTURE based on nuclear markers. The best number of clusters was estimated as two according to the Δ*K* test, whereas the likelihood values “plateaued” from *K* = 2 to *K* = 4 (Fig. S2). For *K* = 2 (Fig. S3), the red cluster primarily comprised *N. incisum*, whereas the second cluster (green one) contained *N*. *franchetii*, *N*. *oviforme*, and *N*. *forrestii* individuals. A second species (*N*. *forrestii*) could be roughly assigned to a single cluster only at *K* = 4, whereas *N*. *franchetii* and *N*. *oviforme* individuals apparently exhibited a pattern of genetic admixture mainly between the green and yellow clusters (Fig. 4 and S3).

**Fig. 5:**
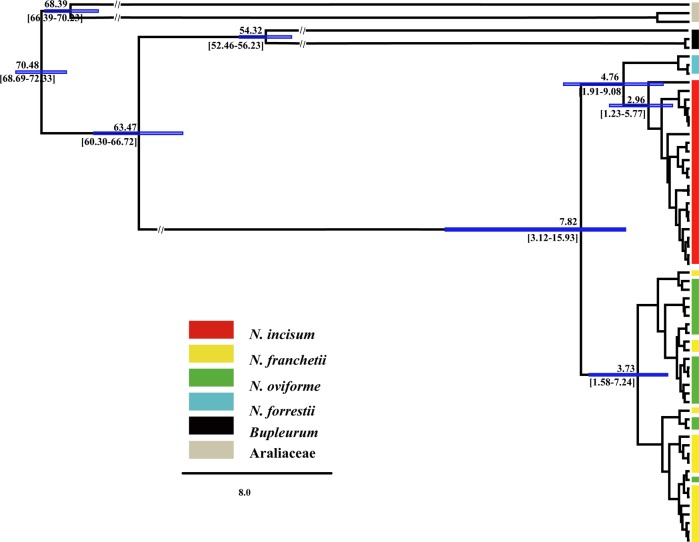
BEAST-derived chronograms of the four studied *Notopterygium* species based on cpDNA haplotypes

The phylogenetic relationships constructed for nuclear loci based on Bayesian methods showed that *N*. *incisum* and *N*. *forrestii* generally constitute monophyletic groups with a high bootstrap value, whereas *N*. *franchetii* and *N*. *oviforme* were polyphyletic in most of the nuclear genes sampled (Fig. S4). This is consistent with the results of genetic structure (*K* = 3 but especially *K* = 4), as *N. incisum* and *N*. *forrestii* formed their own groups, whereas a pattern of strong genetic admixture was found between *N*. *franchetii* and *N*. *oviforme*. The maximum-likelihood phylogenetic analysis based on the concatenated nuclear genes showed a similar topology (Fig. S5). In addition, we estimated the divergence time among the four *Notopterygium* species based on the concatenated cpDNA haplotypes and the concatenated nuclear genes, respectively. The first divergence among the four species occurred ~7.82 Ma [95% HPD (highest posterior density), 3.12–15.93 Ma] based on the cpDNA variation (Fig. 5). With the concatenated nuclear genes, a slightly different crown divergence for the four *Notopterygium* species was obtained, having occurred about 10.90 Ma (95% HPD, 6.74–14.63 Ma; Fig. S6). Interestingly, the 95% HPD largely overlapped between the two analyses. The phylogenetic analysis based on the cpDNA dataset showed that *N*. *incisum* and *N. forrestii* clustered into a genetic lineage, with the divergence between two species having occurred ~4.76 Ma (95% HPD, 1.91–9.08 Ma; Fig. 5). In the nuclear genes tree, *N. forrestii*, *N*. *franchetii*, and *N*. *oviforme* formed a large clade, with the divergence among these species having occurred 7.99 Ma (95% HPD, 4.75–10.96 Ma; Fig. S6).

### Demographic history

We performed all six simulations of species-pair combinations using the IMa2 program (Table 5). The results including the effective population size, divergence time, and migration rate suggested that the IM model captured some general features of the population history for each species. *N. incisum* had the largest estimated effective population size (*N*_e_ = 386,000), whereas *N*. *forrestii* had the lowest one (*N*_e_ = 130,000). The effective sizes of the descendant populations of these four species were ~12–13 times larger than those of the ancestral populations (Table 5), thereby indicating that the four species had all undergone observable population expansions. The divergence times were estimated as 4.42 Ma [90% confidence interval (CI): 2.82–6.67 Ma] between *N*. *incisum* and *N*. *franchetii*, 5.35 Ma (90% CI: 3.59–7.79 Ma) between *N*. *incisum* and *N*. *oviforme*, 6.32 Ma (90% CI: 3.35–11.7 Ma) between *N*. *incisum* and *N*. *forrestii*, 1.74 Ma (90% CI: 0.997–3.08 Ma) between *N*. *franchetii* and *N*. *oviforme*, 4.45 Ma (90% CI: 2.80–6.47 Ma) between *N*. *franchetii* and *N*. *forrestii*, and 4.39 Ma (90% CI: 2.66–6.38 Ma) between *N*. *oviforme* and *N*. *forrestii* (Table 5).

**Table 5:** MLEs and 95% HPD intervals for demographic parameters based on pairwise IMa2 multilocus analyses

Comparison		*θ*_1_	*θ*_2_	*θ*_A_	*m*_1_	*m*_2_	*t*	*N*_1_	*N*_2_	*N*_A_	*T* (years)	2*N*_1_*m*_1_	2*N*_2_*m*_2_
*N. incisum/ N. franchetii*	**MLE**	**0.7250**	**0.5750**	**0.055**	**0.197**	**0.011**	**0.8530**	**3.13** **×** **10**^**5**^	**2.48** **×** **10**^**5**^	**2.37** **×** **10**^**4**^	**4.42** **×** **10**^**6**^	**0.07325**	**0.003578**
	HPD95Lo	0.535	0.405	0.015	0.077	0.0	0.5450	2.31 × 10^5^	1.74 × 10^5^	6.48 × 10^3^	2.82 × 10^6^	0.03009	0.0
	HPD95Hi	0.935	0.775	1.235	0.405	0.179	1.287	4.03 × 10^5^	3.34 × 10^5^	5.33 × 10^5^	6.67 × 10^6^	0.1438	0.04995
*N. incisum/ N. oviforme*	**MLE**	**0.8750**	**0.6350**	**0.005**	**0.103**	**0.001**	**1.033**	**3.77** **×** **10**^**5**^	**2.74** **×** **10**^**5**^	**2.16** **×** **10**^**3**^	**5.35** **×** **10**^**6**^	**0.04583**	**0.0000975**
	HPD95Lo	0.665	0.455	0.0	0.035	0.0	0.6930	2.87 × 10^5^	1.96 × 10^5^	0.0	3.59 × 10^6^	0.01685	0.0
	HPD95Hi	1.105	0.865	1.465	0.223	0.083	1.505	4.77 × 10^5^	3.73 × 10^5^	6.32 × 10^5^	7.79 × 10^6^	0.09623	0.02642
*N. incisum*/*N. forrestii*	**MLE**	**1.085**	**0.2650**	**0.025**	**0.045**	**0.001**	**1.220**	**4.68** **×** **10**^**5**^	**1.14** **×** **10**^**5**^	**1.08** **×** **10**^**4**^	**6.32** **×** **10**^**6**^	**0.02522**	**0.0001475**
	HPD95Lo	0.8350	0.1350	0.015	0.005	0.0	0.6465	3.60 × 10^5^	5.82 × 10^4^	6.48 × 10^3^	3.35 × 10^6^	0.00221	0.0
	HPD95Hi	1.365	0.4650	2.955	0.1370	0.327	2.264	5.89 × 10^5^	2.01 × 10^5^	1.27 × 10^6^	1.17 × 10^7^	0.07183	0.04115
*N. franchetii*/*N. oviforme*	**MLE**	**0.9875**	**0.8725**	**0.1625**	**1.028**	**0.3725**	**0.3365**	**4.26** **×** **10**^**5**^	**3.76** **×** **10**^**5**^	**7.02** **×** **10**^**4**^	**1.74** **×** **10**^**6**^	**0.5232**	**0.1714**
	HPD95Lo	0.7025	0.6175	0.0075	0.3975	0.0	0.1925	3.03 × 10^5^	2.67 × 10^5^	3.24 × 10^3^	9.97 × 10^5^	0.2220	0.0
	HPD95Hi	1.383	1.228	0.5525	2.132	1.058	0.5945	5.97 × 10^5^	5.30 × 10^5^	2.38 × 10^5^	3.08 × 10^6^	1.019	0.4596
*N. franchetii*/*N. forrestii*	**MLE**	**0.7150**	**0.2650**	**0.005**	**0.035**	**0.001**	**0.8590**	**3.08** **×** **10**^**5**^	**1.14** **×** **10**^**5**^	**2.16** **×** **10**^**3**^	**4.45** **×** **10**^**6**^	**0.01334**	**0.0001225**
	HPD95Lo	0.5050	0.1350	0.0	0.0	0.0	0.5410	2.18 × 10^5^	5.83 × 10^4^	0.0	2.80 × 10^6^	0.0	0.0
	HPD95Hi	0.9650	0.4450	1.045	0.1590	0.2690	1.249	4.16 × 10^5^	1.92 × 10^5^	4.51 × 10^5^	6.47 × 10^6^	0.05514	0.03565
*N. oviforme*/*N. forrestii*	**MLE**	**0.8650**	**0.3750**	**0.015**	**0.0295**	**0.0005**	**0.8470**	**3.73** **×** **10**^**5**^	**1.62** **×** **10**^**5**^	**6.48** **×** **10**^**3**^	**4.39** **×** **10**^**6**^	**0.01348**	**0.0001888**
	HPD95Lo	0.5850	0.1950	0.0	0.0	0.0	0.5130	2.52 × 10^5^	8.42 × 10^4^	0.0	2.66 × 10^6^	0.0	0.0
	HPD95Hi	1.195	0.6150	0.9450	0.1635	0.2885	1.231	5.16 × 10^5^	2.65 × 10^5^	4.08 × 10^5^	6.38 × 10^6^	0.06943	0.05379

*θ*_1_, *θ*_2_, *θ*_A_, *m*_1_, *m*_2_, and *t* are scaled by the mutation rate, whereas *N*_1_, *N*_2_, *N*_A_, 2*N*_1_*m*_1_, 2*N*_2_*m*_2_, and *T* are scaled by individuals or years. All estimates include the per gene mutation rate (*μ*), which is equal to the geometric mean of the mutation rate of all loci. *θ*_1_, *N*_1_ effective population size of the first species; *θ*_2_, *N*_2_ effective population size of the second species; *θ*_A_, *N*_A_ effective population size of ancestral population; *HPD* highest posterior density, *m*_1_ population migration rate from the second to the first species; *m*_2_ population migration rate from the first to the second species; MLE maximum-likelihood estimate, *t*, *T* time since species divergence

In addition, the results obtained by ABC analysis (Table S9) clearly favored the hypothetical scenario 4 for *N*. *incisum* and *N*. *franchetii*. In this case, the population contractions have occurred 1.48 Ma and 1.33 Ma, respectively, and the population expansions 64,000 and 47,300 years ago. This hypothesis was consistent with the results of the neutrality test and IM analysis. The best case for *N*. *oviforme* was scenario 1, which implies that a population expansion occurred 214,000 years ago. However, the results for *N*. *forrestii* were somewhat different from those obtained by IM analysis. Scenario 5 was considered to be the best case for *N*. *forrestii*, for which the population contraction occurred 365,000 years ago.

### Ecological niche modeling

**Fig. 6:**
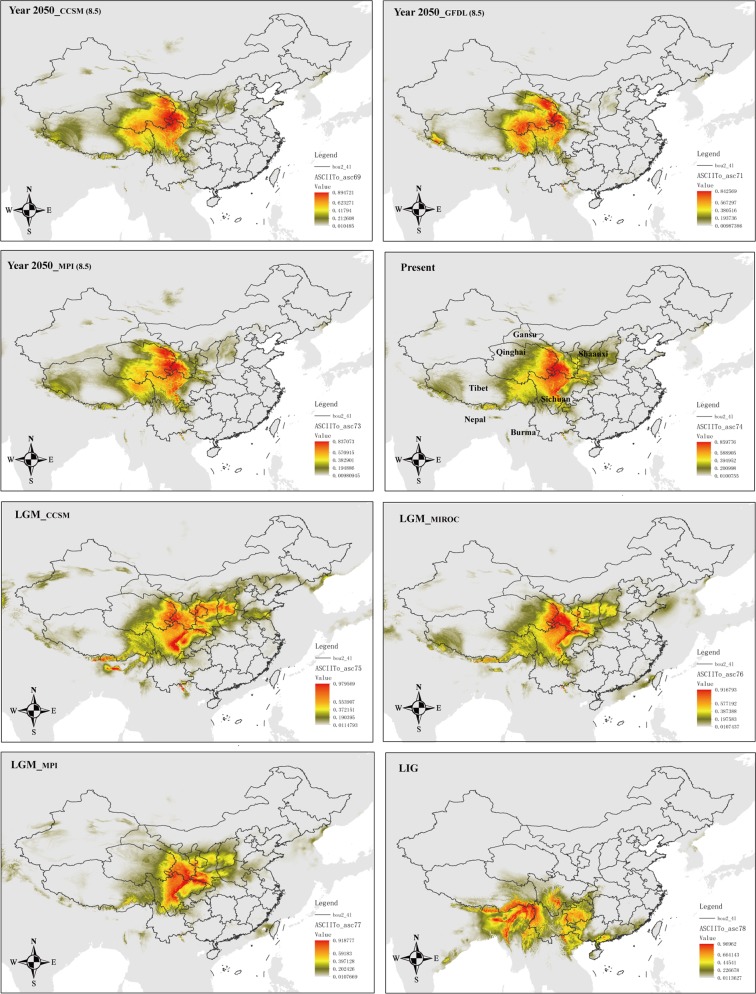
Ecological niche modeling results for *N. incisum* in different periods and with different models. The value of maximum sensitivity plus specificity logistic threshold is 0.1447. Pixels below this value should not be considered as suitable for the species

**Fig. 7:**
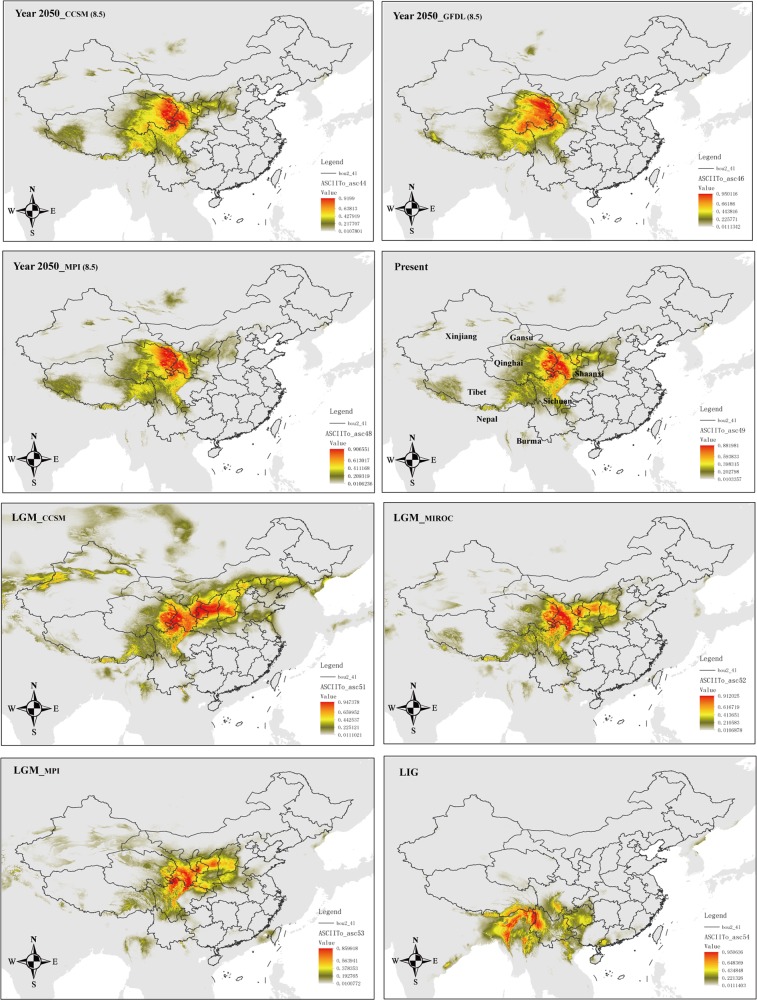
Ecological niche modeling results for *N. franchetii* in different periods and with different models. The value of maximum sensitivity plus specificity logistic threshold is 0.1549. Pixels below this value should not be considered as suitable for the species

**Fig. 8:**
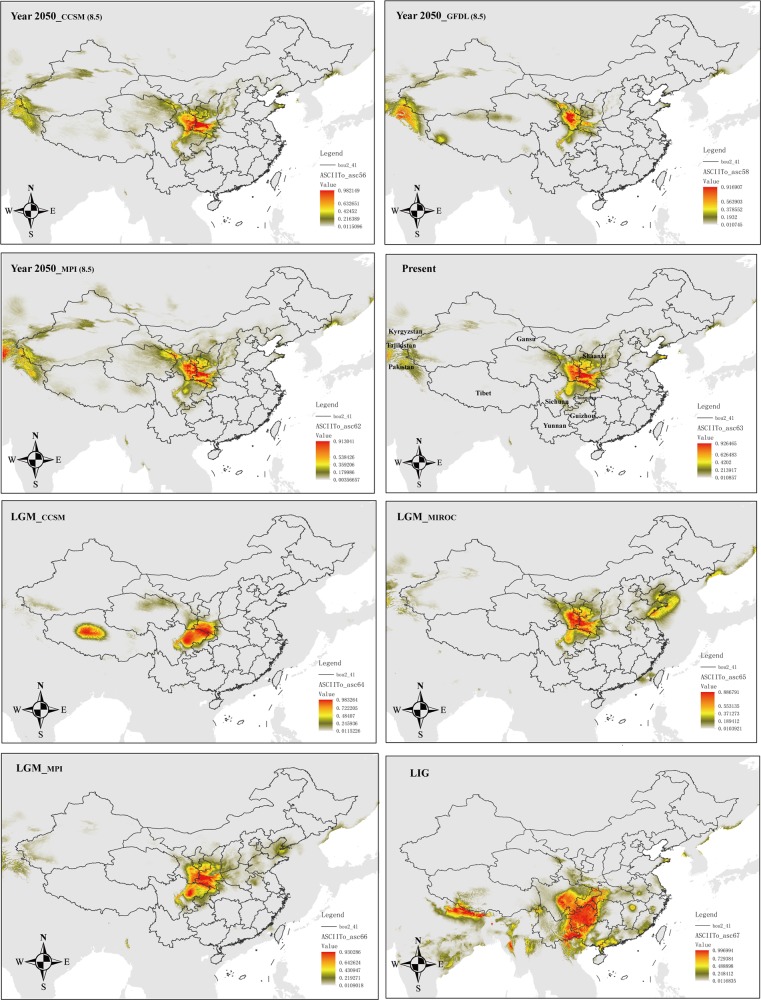
Ecological niche modeling results for *N. oviforme* in different periods and with different models. The value of maximum sensitivity plus specificity logistic threshold is 0.4723. Pixels below this value should not be considered as suitable for the species

We predicted the future (year 2050), current, LGM, and LIG distributions for *N*. *incisum*, *N*. *franchetii*, and *N*. *oviforme* as shown in Figs. 6–8 and Fig. S7. According to ENM results, during the LIG *N*. *incisum* would have occurred mainly in the southwest of China (southern Sichuan, Guizhou, northwestern Yunnan, and eastern Tibet) but also out of current Chinese borders including Myanmar and NE India (Fig. 6). In the three models of LGM, the potential distribution range of *N*. *incisum* would have expanded and moved northward compared with the LIG, occupying most of Sichuan, Shaanxi, eastern Qinghai, southern Gansu, southern Shanxi, and eastern Tibet. From the LGM to the present period, *N*. *incisum* persisted in more or less the same areas (perhaps with a slight westward movement) but it underwent a slight contraction. In the future, the six assayed scenarios showed similar distributions among them, with the potential species range remaining superficially unchanged compared with the present (at most, there is a slight expansion toward the west; Fig. 6 and S7). The same general pattern was found for *N*. *franchetii* (Fig. 7 and S7), with an expansion and northeastward migration from the LIG to the LGM, followed by a relative stable distribution for the period present–2050 (but with a westward shift compared with the LGM). It should be noted, however, that the CCSM model for the LGM of *N. franchetii* showed a considerable larger potential area than the other two LGM models, including a continuous strip of suitable area from Qinghai to Liaoning (Fig. 7). We determined a very different pattern for the changes in the distribution of *N. oviforme* according to the models (Fig. 8 and S7). Thus, starting with a widespread distribution during the LIG period in central-southern China (eastern Sichuan, Chongqing, Guizhou, western Hubei, eastern Yunnan, and western Guangxi), it underwent a very severe range contraction by the LGM, but with small differences depending on the model: although the three LGM models showed that the main range of the species would have been around the confluence of Gansu, Shaanxi, and Sichuan provinces, the CCSM model had an area of suitability in western Tibet, whereas the MIROC model showed that coastal areas of Shandong and contiguous exposed areas would have been adequate for *N. oviforme*. From the LGM to the present and to the year 2050, the distribution of *N*. *oviforme* in central China was more or less maintained (perhaps with a slight contraction for the year 2050), coupled with the appearance of an area of suitability in the border region of Xinjiang with several central Asian countries (Fig. 8 and Fig. S7).

### Climatic niche comparisons

The PCA-env represented 84.15% (PC1 = 52.81% and PC2 = 31.34%) of the total climatic variation occupied by the species and background areas (Fig. S8). As the main contributing variables to the principal components, bio1 (annual mean temperature) was the first in the PC1 and bio12 (annual precipitation) in the PC2. The most distant climatic niche respect to the others was that of *N. oviforme* (Fig. S8 and *D*_S_ values in Table S10), especially when only the 20% of occurrence density was displayed (Fig. S8B). The niche equivalency test confirmed a niche divergence between *N. oviforme* and *N. franchetii* (Table S10). In addition, we also detected that *N. oviforme* is occupying two distinct niches in climatic space. The species *N. incisum* and *N. franchetii* showed high levels of climatic niche overlapping in the PCA-env and regarding stability values (Table S10), although the occurrence density clouds were slightly separated along the PC1 even sharing almost equal available background climates (dashed lines in Fig. S8C). In similar way, although equivalency test did not result in a significant niche conservatism pattern (i.e., niches were not interchangeable), the similarity test—which takes into account surrounding available areas—revealed that *N. incisum* and *N. franchetii* niches were more similar than expected by chance (Table S10). The highest values of niche expansion respect to the rest were attributed to *N. incisum* (Table S10).

## Discussion

### Genetic variation and population structure

The nucleotide variation at silent sites is basically consistent with neutral expectations of molecular evolution; thus, it can be considered as a good surrogate for the genetic diversity at the species level (Li et al. 2012). To obtain accurate estimations of population genetic parameters for the four *Notopterygium* species, we randomly selected some orthologous nuclear genes by comparative transcriptome analysis of *N*. *franchetii* and *N*. *incisum* (Jia et al. 2017). We found that the average silent nucleotide diversity value (*π*_s_) at ten nuclear loci for *N*. *oviforme* (0.0055) is higher than those for *N*. *franchetii* (0.0050), *N*. *incisum* (0.0036), and *N*. *forrestii* (0.0029). The average total nucleotide diversity is also slightly higher in *N*. *oviforme* (*π*_t_ = 0.0035) than in *N*. *incisum* (*π*_t_ = 0.0031), *N*. *franchetii* (*π*_t_ = 0.0027), and *N*. *forrestii* (*π*_t_ = 0.0023). These values are comparable to the levels of diversity in Umbelliferae herbs and higher than those in other alpine herbaceous plants, e.g., *Cardamine nipponica* Franch. and Sav. (Brassicaceae): *π*_t_ = 0.0011–0.0015 (Ikeda et al. 2009); *Libanotis buchtormensis* Fisch (DC.) (Umbelliferae): *π*_t_ = 0.0033–0.0037 (Wang et al. 2016b); *Oxyria digyna* (L.) Hill (Polygonaceae): *π*_t_ = 0.0007–0.0032 (Wang et al. 2016a); *Rhodiola chrysanthemifolia* (H. Lév.) S. H. Fu (Crassulaceae): *π*_t_ = 0–0.0016 (Gao et al. 2016); and *Trailliaedoxa gracilis* W.W. Sm. and Forrest (Rubiaceae): *π*_t_ = 0–0.0002 (Jia et al. 2016).

In general, the four *Notopterygium* species investigated in the present study are mostly distributed in the eastern part of the QTP (Hengduan Mountains sensu lato plus Qingling Mountains) and their high genetic diversity is consistent with the role of these mountains as Pleistocene refugia (Huang et al. 2015; López-Pujol et al. 2011). Large parts of the Hengduan Mountains, as well as the Qingling Mountains and other ranges in central China, were never glaciated and their diverse topography provided pockets with sufficient eco-climatic stability where populations maintained relatively large sizes, and thus continued gene flow throughout the glacial/interglacial cycles (Zhang et al. 2018). Similar to the nuclear markers (see above), *N*. *oviforme* has the highest levels of total genetic diversity based on cpDNA, i.e., *H*_T_ = 0.910, followed by *N*. *incisum* (*H*_T_ = 0.801) and *N*. *franchetii* (*H*_T_ = 0.371). These diversity values are also similar with the previously reported results (*N*. *oviforme* *H*_T_ = 0.961, *N*. *incisum* *H*_T_ = 0.939, and *N*. *franchetii* *H*_T_ = 0.766) based on the three cpDNA fragments (*trnS-trnG*, *matK*, and *rbcL*) (Shahzad et al. 2017). Assuming that the four *Notopterygium* species share similar life-history traits, then the higher genetic levels in *N*. *oviforme* might be explained by the fact that most of its range is located in the Qinling Mountains and in the low-altitude mountain ranges of central and western China, far from the colder and drier (at present but also during the LGM; Tian and Jiang 2016) areas of western Sichuan and northeastern Qinghai (and the adjacent areas of Gansu), where most of the populations of *N*. *incisum* and *N*. *franchetii* are located. Among the *Notopterygium* species considered in this study, *N*. *forrestii* has the lowest levels of genetic diversity, as expected for a species with such a small distribution area (Hamrick and Godt 1990; Nybom 2004). The ecological heterogeneity among the different distributional regions might have been responsible, therefore, for the different levels of genetic diversity of the natural populations of the four *Notopterygium* species.

The population genetic structure may be affected mostly by dynamics of climate and landscape across the evolutionary history of populations (McDonald and Hamrick 1996; Potter et al. 2015). In this study, AMOVA shows that most of the genetic variation detected based on cpDNA and all nuclear genes is found among species (*P* < 0.001), whereas the genetic variation in mtDNA is due mainly to differences among populations within species. These differences might be attributable to the limited number of informative sites and the slow evolutionary rate in the mtDNA markers (Palmer and Herbon 1988). Despite these differences, the AMOVA patterns are, in general, congruent with species that diverged long time ago, and that occur in a region with very rugged terrain (the Hengduan Mountains are highly dissected, with elevation gradients of over 3000 m; Boufford 2014). Indeed, the cpDNA sequences of the four *Notopterygium* species indicate a high level of differentiation among species (*G*_ST_ = 0.790 for the four species as a whole; Table 3) and the individual values (0.717, 0.774, and 0.522 for *N*. *incisum*, *N*. *oviforme*, and *N*. *franchetii*, respectively) were even higher than the *G*_ST_ values reported for other QTP endemic alpine herbs, including *Pomatosace filicula* Maxim. (*G*_ST_ = 0.518) (Wang et al. 2014b) and *L.buchtormensis* (*G*_ST_ = 0.671) (Wang et al. 2016b). The general *G*_ST_ value is significantly smaller than *N*_ST_ for *Notopterygium* (0.790 vs. 0.959; Table 3), thereby suggesting that there is phylogeographic structure. When *G*_ST_ and *N*_ST_ are compared within each species, only for *N*. *oviforme* there is no signal of phylogeographic structure despite that *N*_ST_ is greater than *G*_ST_ (0.817 vs. 0.774). The existence of a significant geographic genetic structure for most of *Notopterygium* species may be associated with the heterogeneous environments inhabited by their natural populations in the QTP and the associated mountains in its eastern margin.

### Species divergence

The uplift of the QTP since the Cenozoic had significant impacts on the differentiation and genetic structure of plant species (Qiu et al. 2011; Xing and Ree 2017). Although some studies have shown that the main uplift of the QTP occurred about 8–10 Ma (Molnar et al. 1993) or even in more recent times (Li and Fang 1999), there is a growing consensus that the plateau underwent a relative constant uplift since 40 Ma (Favre et al. 2015; Mulch and Chamberlain 2006). Recently, Renner’s (2016) suggested that the QTP would have reached 4–5 km high even since the mid-Eocene (about 40 Ma). Some studies, however, have shown that the eastern margin of the QTP (where most of the studied populations of *Notopterygium* are located) started to uplift later, probably after 10 Ma (Favre et al. 2015; Xing and Ree 2017), and the highest elevation was probably reached just before the late Pliocene (Sun et al. 2011). Molecular dating based on the cpDNA variation shows that the four *Notopterygium* species have initially diverged ~7.82 Ma (Fig. 5), whereas that based on nuclear genes indicates that this occurred somewhat before, about 10.90 Ma (Fig. S6), although the 95% HPD are largely overlapping (3.12–15.93 Ma and 6.74–14.63 Ma for cpDNA and nuclear loci, respectively). Similarly, our IM analysis suggests that the divergence of *Notopterygium* species probably occurred during the late Miocene. The split between *N*. *incisum* and *N*. *forrestii*, which was the earliest within our study species, can be placed at 6.32 Ma (95% HPD: 3.35–11.7 Ma). The other divergence events (with the exception of the split between *N*. *franchetii* and *N*. *oviforme*) occurred during the following two million years (Table 5), i.e., within the late Miocene and early Pliocene. Although we must be cautious regarding the results of molecular dating, these divergence time estimates coincide with a period of intense uplift of the Hengduan Mountain massif (Favre et al. 2015; Sun et al. 2011), which may have produced many small fragmented habitats with different microclimates, thereby impacting the direction of natural selection (Sobel et al. 2010). Indeed, the uplift of the QTP had a great impact on the climate in China but also on the whole of Asia (An et al. 2001), including temperature decreases in some areas. These new climatic conditions may have been conducive to the expansion of populations of cold-resistant plants. Furthermore, numerous studies of other herbs, shrubs, and animal groups have generated dated molecular phylogenies that indicate the occurrence of extensive species diversification in the QTP and adjacent regions during the Pliocene (e.g., Liu et al. 2002; Jia et al. 2012; Xu et al. 2010; Zhou et al. 2012). The latest species divergence within *Notopterygium* was between *N*. *oviforme* and *N*. *franchetii* at 1.74 Ma (95% HPD: 0.997–3.08 Ma), which closely corresponds with the largest glaciations of the early–middle Pleistocene in this region (0.5–1.2 Ma) and the deformed uplift of the QTP at ca. 1.6–3.6 Ma (Liu et al. 2014; Zheng et al. 2002). Therefore, it is likely to be that fragmentation of the species’ distributions caused by QTP uplift may have promoted intraspecific and interspecific divergence on a large scale in the region. In addition, niche differentiation among species in diverse environments in the QTP and adjacent areas may have influenced divergence; although the niche of *N. oviforme* has almost completed its divergence, niches of *N. franchetii* and *N. incisum* might have initiated such process (Fig. S8).

IM analysis provided unambiguous evidence of the interspecific bidirectional asymmetric gene flow among these four species, which was consistent with the STRUCTURE clustering results. Gene flow from *N*. *incisum* to *N*. *franchetii*, *N*. *oviforme*, and *N*. *forrestii* was much lower than that in the opposite direction (i.e., to *N*. *incisum*). Similarly, the gene flow from *N. forrestii* to *N*. *franchetii* and *N. oviforme* was much higher than that from *N*. *franchetii* and *N. oviforme* to *N. forrestii* (Table 5). This pattern might be explained by the fact that *N*. *incisum* and *N*. *forrestii* were the earliest differentiated species. In contrast, *N*. *franchetii*−*N*. *oviforme* is the only species pair with relatively comparable levels of gene flow for the two directions; in addition, gene flow between *N*. *franchetii* and *N*. *oviforme* is by far the highest among all species pairs (with differences of one order of magnitude; Table 5) and this was probably because these are the most recently diverged species. The parapatric geographic distribution of *N*. *franchetii* and *N*. *oviforme* may have provided the opportunity for interspecific gene flow and hybridization among them. Interestingly, the STRUCTURE analysis also shows that a large amount of genetic variation is shared among the four species, especially between *N. franchetii* and *N. oviforme* (which are almost genetically indistinguishable when *K* *=* 2, 3, and 4). In addition, the incongruent phylogenetic topologies of cpDNA vs. nuclear genes should also be noted, which could be signals of hybridization and/or backcrosses between these species (Kim and Donoghue, 2008; Sang and Zhong, 2000). According to field observations, we found that these two species have overlapping flowering times, which may have facilitated genetic introgression and/or hybridization. Previous studies have also suggested the occurrence of hybridization among tree species distributed in the same geographic regions and subsequent backcrosses with one of the parental species, and these processes resulted in high levels of shared genotypes (Hamzeh et al. 2006; Li et al. 2013; Wang et al. 2014a). In addition, we should not completely exclude the presence of incomplete lineage sorting due to the recently species divergence among these *Notopterygium* species, which might have caused the sharing of interspecific genetic polymorphisms.

### Demographic history

It is generally assumed that alpine plants have experienced important distributional shifts driven by glacial/interglacial cycles (Hewitt 2000). The current ABC analysis results suggest that two of the four *Notopterygium* species, *N*. *incisum* and *N*. *franchetii*, have experienced an earlier population contraction, followed by a recent population expansion. The results of such earlier population contraction are also supported by the negative average values of neutral tests, e.g., Tajima’s *D* and Fay and Wu’s *H* statistics (Haddrill et al. 2005). The bottleneck and population contraction of the two *Notopterygium* species could be related to the uplift of the QTP at 1.6–3.6 Ma (Liu et al. 2014). The uplift of the plateau may have caused fragmentation of the habitat, and the unfavorable environment may have led to the extinction of some populations while producing population contraction in others. In terms of the recent expansion of the populations, previous studies found that the QTP experienced, during the period 30–70 Ka, a stadial (a colder stage) during the last glacial period (Shi 2002; Zheng et al. 2002). However, the *Notopterygium* species are well adapted to cold climatic conditions, such stadial would have been beneficial for the expansion of their populations. The ENM results also support this view, as in both *N*. *incisum* and *N*. *franchetii* there is a range expansion from the LIG to the LGM (Figs. 6 and 7). Regarding the other two studied *Notopterygium* species, the ABC analysis suggests that *N*. *oviforme* experienced a population expansion about 214,000 years ago, whereas *N*. *forrestii* suffered a population contraction ca. 365,000 years ago. Such demographic events could be linked to the glacial/interglacial cyclicity recorded in the QTP, as the antepenultimate interglacial period and the penultimate glacial period took place between 500 and 300 Ka, and between 300 and 130 Ka, respectively (Zheng et al. 2002). However, these inferences should be treated with extreme caution given their large CIs (Table S9). The glacial climatic conditions were not inherently unfavorable or restrictive for all plant species in our study area. Some cold-tolerant species with a wide range of habitats and vegetation zones certainly survived in multiple refugia on the QTP throughout glacial/interglacial periods, such as *A. gymnandrum* (Wang et al. 2009a), *P. glabra* (Wang et al. 2009b), *Juniperus tibetica* Kom. (Opgenoorth et al. 2010), and *Rhodiola alsia* (Fröd.) S. H. Fu (Gao et al. 2009).

In conclusion, alpine herb plants in the high-altitude QTP may have experienced different demographic histories. Some studies, as reported here, have shown that alpine plant species were more widely distributed and interconnected during glacial periods but they became fragmented during the warm interglacials due to the retreat of the ice (Rutherford and D’Hondt 2000; Winograd et al. 1997). Therefore, the herbs that at present are found in cold environments may have exhibited different population dynamics during past climatic oscillations compared with species associated with warmer environments. Thus, our findings highlight the importance of geological and climatic changes during the Miocene–Pliocene but also Pleistocene as drivers of species divergence and changes in population structure within cold-tolerant herbal species in the QTP biodiversity hotspot. It is also remarkable that the range of the three species whose niche has been modeled for the year 2050 (*N*. *incisum*, *N*. *franchetii*, and *N*. *oviforme*) do not change significantly compared to the present time; even, a small increase can be inferred for the first two species (with a somewhat westwards expansion). Such pattern, contrary to what one might expect, has been widely reported for a series of subalpine and alpine plants of the Hengduan Mountains and the same reasons provided by the authors of such study can be advocated here: that the large adjacent mountain ranges of the QTP (toward the west) might have had an important role as an escape region (Liang et al. 2018).

### Data archiving

Nucleotide data have been deposited at NCBI (https://www.ncbi.nlm.nih.gov/nucleotide/) under the following Accession IDs MK312210–MK312239, MK305312–MK305813, and MK258173–MK258185.

## Supplementary information

Supplemental material info

Supplementary material
