# Supplementary material for: Complex population evolutionary history of four cold-tolerant Notopterygium herb species in the Qinghai-Tibetan Plateau and adjacent areas
Source: Heredity (Edinb). 2019 Feb 11;123(2):242–63. doi: 10.1038/s41437-019-0186-2 (PMC6781143; doi:10.1038/s41437-019-0186-2)
Supplement: Supplementary file 1 — Supplemental material info [file 41437_2019_186_MOESM1_ESM.docx]

Supplementary materials

Table S1 List of sampled populations of *Notopterygium incisum*, *N. franchetii*, *N. oviforme*, and *N. forrestii*, with information on their locations (geographic coordinates, altitude), sample size (*N*), and voucher number.

Table S2 Details of primers used in this study.

Table S3 Parameters used as prior settings in DIYABC analysis.

Table S4 Details of the 19 ecological variables employed in this study.

Table S5 Sampling location information in ecological niche modeling (ENM).

Table S6 Gene diversity, nucleotide diversity, and haplotype frequencies of the cpDNA and mtDNA sequences for all studied populations of four *Notopterygium* species. *N*, number of samples; *θ*_wt_ , Watterson’s *θ*; *π*_t_, total nucleotide diversity. Note: H(a) b means that haplotype “a” is shown by “b” individuals.

Table S7 Analysis of molecular variance (AMOVA) based on variation at 10 nuclear loci, cpDNA, and mtDNA in four species of *Notopterygium*.

Table S8 Genetic differentiation at 10 nuclear loci among: (a) *Notopterygium incisum*, (b) *N. franchetii*, (c) *N. oviforme*, and (d) *N. forrestii*.

Table S9 Estimated posterior distributions of the parameters according to Approximate Bayesian Computation (ABC) for the best scenario of the demographic history for the four *Notopterygium* species.

Table S10 Comparisons of the current occupied climatic niches by pairs (*A* vs. *B*) of three *Notopterygium* species, based on E-space analysis. Schoener’s *D*_S_ represents niche overlap between niches (*D*_S_; 0 = no overlap, 1 = complete overlap). The niche equivalency (eq) and niche similarity (sim) tests are considered significant (*P* < 0.05, showed in bold) when niche overlap is lower than randomly expected (niche divergence; D), or larger than randomly expected (niche conservatism; C). The niche unfilling, stability, and expansion are also presented. Not applicable cases are shown as NA.

Figure S1 Haplotype (= allele) genealogies at 10 nuclear loci in the four studied *Notopterygium* species.

Figure S2 Two methods used to estimate the best *K* values with STRUCTURE based on nuclear markers, in the four studied *Notopterygium* species.

Figure S3 STRUCTURE analysis of four *Notopterygium* species when *K* = 2–4 clusters were assumed.

Figure S4 Bayesian inference phylogenetic trees based on each of the 10 single copy nuclear loci, in the four studied *Notopterygium* species.

Figure S5 The Maximum Likelihood phylogenetic tree based on concatenated 10 single copy nuclear loci, in the four studied *Notopterygium* species.

Figure S6 BEAST-derived chronograms of four species based on concatenated 10 single copy nuclear loci, in the four studied *Notopterygium* species.

Figure S7 Species distribution modeling results for *N. incisum*, *N. franchetii* and *N. oviforme* for the year 2050 using three models: CCSM, GFDL and MPI based on RCP 2.6.

Figure S8 (A) Geographic location of the occurrence records and background areas used for the climatic niche comparison in E-space of three *Notopterygium* species: *N. franchetii* (blue), *N. incisum* (yellow), and *N. oviforme* (red).
