# Supplementary material for: Complex population evolutionary history of four cold-tolerant Notopterygium herb species in the Qinghai-Tibetan Plateau and adjacent areas
Source: Heredity (Edinb). 2019 Feb 11;123(2):242–63. doi: 10.1038/s41437-019-0186-2 (PMC6781143; doi:10.1038/s41437-019-0186-2)
Supplement: Supplementary file 2 — Supplementary material [file 41437_2019_186_MOESM2_ESM.pdf]

Table S1 List of sampled populations of *Notopterygium incisum*, *N. franchetii*, *N. oviforme*, and *N. forrestii*, with information on their locations (geographic coordinates, altitude), sample size (*N*), and voucher number.

Species	Population	Location	Latitude	Longitude	Altitude (m)	*N*	Voucher number
	A	Huzhu, Qinghai	102.4319	36.8918	2620	6	WNULZH20130807
	B	Maqu, Gansu	102.0703	33.9992	3479	3	WNULZH20130816
	C	Datong, Qinghai	101.8527	37.1496	3030	5	WNULZH20130809
	D	Taibaishan, Shaanxi	108.7797	33.8532	2800	6	WNULZH20140814
	E	Guangtoushan, Shaanxi	107.7010	34.0535	3190	5	WNULZH20140823
	G	Jiuzhi, Qinghai	101.6890	32.8584	4030	5	WNULZH20140821
	H	Maqin, Qinghai	100.1971	34.4904	4030	6	WNULZH20140824
	I	Tongde, Qinghai	100.5467	35.2760	3259	6	WNULZH20130822
	J	Aba, Sichuan	101.0998	33.3834	4030	5	WNULZH20130729
	L	Kangding, Sichuan	101.9669	29.9889	3560	3	WNUJY20150915
N. incisum	N	Yajiang, Sichuan	101.3272	30.0611	4000	5	WNUJY20150910
(*N* = 123)	Q	Yaan, Sichuan	102.8176	30.3683	3450	5	WNUJY20150903
	S	Muli, Sichuan	100.6510	28.2637	3750	4	WNUJY20150720
	T	Qinglin, Qinghai	101.5308	37.3207	3200	2	WNULZH20150718
	U	Daofu, Sichuan	101.3827	31.4693	3920	4	WNULZH20150722
	V	Xiaojinxian, Sichuan	102.6387	32.1214	3219	5	WNUFWB20160721
	W	Xiaojinxian, Sichuan	102.7960	32.2397	3900	4	WNUFWB20160725
	X	Ganzi, Sichuan	100.4784	32.3010	4073	5	WNUFWB20160727
	Y	Luhuo, Sichuan	101.5596	31.8943	3465	5	WNUFWB20160719
	Z	Yuzhong, Gansu	104.3609	35.7667	3046	5	WNULZH20130717
	HA	Tianzhu, Gansu	103.2543	37.9120	3102	5	WNUJY20150710
	HB	Danba, Sichuan	102.1853	30.9336	3708	5	WNULZH20130912

	HC	Maerkang, Sichuan	103.3876	32.7876	4652	5	WNUJY20151106
	HF	Taibaishan, Shaanxi	108.2255	34.9387	3323	5	WNUJY20151203
	HH	Zhouqu, Gansu	104.5107	34.1207	3360	5	WNULZH20130807
	HI	Datong, Qinghai	102.2979	38.1388	3150	5	WNULZH20130812
	KA	Huzhu, Qinghai	102.4319	36.8918	2110	6	WNULZH20140903
	KC	Hepingzhen, Gansu	103.9551	36.0039	2450	6	WNULZH20140805
	KD	Minhe, Qinghai	102.7054	36.0847	2450	6	WNULZH20130915
	KF	Yuzhong, Gansu	104.0576	35.7966	2484	6	WNULZH20130809
	KG	Yaan, Sichuan	102.8176	30.3683	2890	5	WNULZH20140816
	KH	Datong, Qinghai	101.8527	37.1496	2319	6	WNULZH20140824
	KI	Maqu, Gansu	102.0703	33.9992	2379	5	WNULZH20140817
	KJ	Tongde,Qinghai	100.5467	35.2760	2273	6	WNULZH201340910
	KK	Nuoergai, Sichuan	102.9615	33.5903	3526	6	WNULZH20130903
N. franchetii	KL	Datong, Qinghai	101.5308	37.3207	3200	5	WNUJY20150706
(*N* = 135)	KM	Yundingshan, Shanxi	111.5310	37.8906	2543	5	WNUJY20151106
	KO	Zhangye, Gansu	101.4667	38.7167	2800	5	WNULZH20140801
	KQ	Yuzhong, Gansu	104.0375	35.7778	2400	4	WNULZH20140802
	KR	Dangchang, Gansu	104.2590	34.2257	2470	3	WNULZH20130406
	KV	Hezheng, Gansu	103.3487	35.4249	2143	4	WNULZH20131110
	KX	Datong, Qinghai	101.4009	37.0841	2058	6	WNUJY20150731
	KZ	Jishishan, Gansu	102.8741	35.7181	2281	6	WNUJY20150725
	YA	Weiyuan, Gansu	103.9837	35.1236	2934	5	WNUJY20150720
	YB	Yuzhong, Gansu	104.6745	35.3105	2847	5	WNUJY20150721
	YC	Daofu, Sichuan	101.3204	31.8563	3189	5	WNULZH2015071
	YD	Danba, Sichuan	102.2000	30.5667	3318	4	WNULZH20150727
	YE	Tianzhu, Gansu	103.4026	37.5991	2816	5	WNUJY20150721

	YF	Luhuo, Sichuan	101.2373	31.8869	3246	5	WNUJY20150804
	YH	Zhouqu, Gansu	105.0310	33.6443	1823	5	WNULZH20150410
	YJ	Pingan, Qinghai	102.8747	36.7249	2690	5	WNULZH20150912
	YK	Datong, Qinghai	102.3506	37.1864	3058	3	WNUFWB20160911
	YM	Fuzhong, Qinghai	102.2593	37.1722	2958	3	WNUFWB20160721
	LA	Taibaishan, Shaanxi	107.7011	34.0535	3190	5	WNUFWB20160819
	LB	Xihuazhen, Gansu	106.5856	35.1610	2650	4	WNUFWB20160824
	LC	Longxian, Shaanxi	106.6734	35.0690	2568	5	WNULZH20130704
	LD	Zhuque Forest Park, Shaanxi	108.5268	33.9248	1890	4	WNUJY20150519
	LE	Jiwozi, Shaanxi	108.8230	33.8205	2430	4	WNUJY20150717
	LF	Gangu, Gansu	105.1848	34.5744	2234	2	WNUJY20151001
	LG	Xihuazhen, Gansu	106.5821	35.1609	2480	5	WNULZH20150704
N. oviforme	LK	Fengyukou, Shaanxi	108.7230	33.7205	2300	4	WNULZH20150730
(*N* = 66)	LO	Xunyangba, Shaanxi	109.0716	34.4095	2410	5	WNUFWB20160728
	LP	Longxian, Shaanxi	107.1761	35.5032	2153	4	WNUFWB20160722
	LQ	Fengyukou, Shaanxi	108.6230	33.6205	2300	5	WNUFWB20160804
	LR	Jiwozi, Shaanxi	108.8230	33.8205	2430	5	WNUFWB20160812
	LT	Guanshan, Gansu	106.4023	35.1588	2427	4	WNULZH20130817
	LU	Guanshan, Gansu	106.6532	35.2183	2387	5	WNUJY20150708
	LV	Zhuque Forest Park, Shaanxi	108.5268	33.9248	1890	5	WNULZH20130726
	LQA	Yajiang, Sichuan	100.5662	30.1583	4164	5	WNUJY20150728
N. forrestii	LQB	Yajiang, Sichuan	100.7859	30.0441	4220	3	WNULZH20130729
(*N* = 16)	LQC	Litang, Sichuan	100.3092	29.9981	4010	4	WNUJY20150724
	LQD	Kalazishan, Sichuan	100.6326	30.1369	4300	4	WNULZH20130726

Table S2 Details of primers used in this study.

Primer		Primer sequence (5'－3')	Annealing temperature	Gene/Gene product
35	F:	GATTGGTCGGATGGAAAAGG	60.0 °C	Pentatricopeptide repeat-containing protein At3g15200
	R:	GGCACCAGACCCTTCAATGT		
OG29101	F:	TTTTAGTTCGCTCACCTGCC	51.2 °C	Pre-mRNA-splicing factor ATP-dependent RNA helicase DEAH1-like
	R:	CCAACTTCTCAACTCAGTGGT		
9122	F:	TCTCATTAGAAAAACCATCCA	56.9 °C	Calcium-binding protein CML47
	R:	ATGATTCTCAATCCTTGCATA		
25629	F:	TTGGTTTTCTACTTGGGCTT	54.6 °C	Serine/threonine receptor-like kinase NFP
	R:	TTGGTCTTGGACAACTTTCA		
25679	F:	AAAGTTCAGTACGGACGCTGT	55.0 °C	Hypothetical protein
	R:	ACTCCCTATGGCTCTTGTTTG		
29206	F:	ACCTTCTCCGAAACCTACCAAT	58.0 °C	Glutathione S-transferase
	R:	TAGCCGAAGAGTTGAAATTGCT		
32125	F:	CAGTTGAAAGTCCTGAGCAGT	52.0 °C	Hypothetical protein
	R:	GTGTGGTTTGATTATCGGTCG		
51964	F:	TTGTCAGATCTATTCGTTCTCG	58.0 °C	50S ribosomal protein L7/L12
	R:	TCAGATAGGCAACAGTCTTCAG		
OG29960	F:	TGTAGTCATTCAGGTTTAGTCAG	59.2 °C	Pentatricopeptide repeat-containing protein At5g13270
	R:	AGTCTCTCGCTGTGGTCAAG		
OG29988	F:	TGAAAAAATCCCTACCAGC	54.6 °C	Nuclear transcription factor Y subunit B-3-like
	R:	AGTAGGTGAGTTAGTAGTATCCA		
OG28079 (cpDNA)	F:	GAAGAAGTCCCGCTCCTATTA	52.0 °C	*ndhF*
	R:	AAGTTGGTCATATCATTTGCC		
OG537 (mtDNA)	F:	TTTCTGGTTTACACGACTTCAGGC	55.0 °C	*nad5*
	R:	CCATCTTCCGCATATCTTGCTCAT		
OG917 (mtDNA)	F:	CCCCAACTCAATCAATATCA	53.5 °C	*orf*33-*nad1*
	R:	GGGAACCATCACAAGTACGG		

Table S3 Parameters used as prior settings in DIYABC analysis.

Parameter	Scenario		
	**Distribution**	**Minimum**	**Maximum**
Effective population size			
NA	uniform	100	1000000
N1	uniform	100	1000000
N2	uniform	100	1000000
N3	uniform	100	1000000
N4	uniform	100	1000000
N5	uniform	100	1000000
Time of events			
t1	uniform	1000	500000
t2	uniform	5000	1000000
Mean mutation rate uniform	uniform	6.1×10^{-9}	1.5×10^{-8}

Table S4 Details of the 19 ecological variables employed in this study.

**Variables**	
Bio1	Annual Mean Temperature
Bio2	Mean Monthly Temperature Range
Bio3	Isothermality (bio2/bio7) (*100)
Bio4	Temperature Seasonality (standard deviation *100)
Bio5	Max. Temperature of Warmest Month
Bio6	Min. Temperature of Coldest Month
Bio7	Temperature Annual Range (bio5 – bio6)
Bio8	Mean Temperature of Wettest Quarter
Bio9	Mean Temperature of Driest Quarter
Bio10	Mean Temperature of Warmest Quarter
Bio11	Mean Temperature of Coldest Quarter
Bio12	Annual precipitation
Bio13	Precipitation of Wettest Month
Bio14	Precipitation of Driest Month
Bio15	Precipitation Seasonality (Coefficient of Variation)
Bio16	Precipitation of Wettest Quarter
Bio17	Precipitation of Driest Quarter
Bio18	Precipitation of Warmest Quarter
Bio19	Precipitation of Coldest Quarter

Table S5 Sampling location information in ecological niche modeling (ENM). CVH (Chinese Virtual Herbarium), NSII (National Specimen Information Infrastructure).

Species	Longitude	Latitude	Accession number	Collectors	Date	Location (Province, locality)	Source	Reliability & accuracy
	95.1408	34.8768	-	-	-	Qinghai, Qumalaixian	CVH	Confirmed
	96.6721	32.9226	19960829	T.N. Ho	1996-08-29	Qinghai, Nongqie	NSII	Confirmed
	97.0020	33.9351	19960814	T.N. Ho	1996-08-14		NSII	Confirmed
	97.0133	33.0062	19710708	Benzaho Guo	1971-07-08	Qinghai, Yushu	NSII	Confirmed
	98.2050	33.1876	-	-	-	Sichuan, Shiquxian	NSII	Confirmed
	98.9675	32.0594	WUK0300999	Yeqi He	1976-08-28	Sichuan, Queer Mountain	CVH	Confirmed
	99.3268	38.9205	PE00931566	-	1964-07-24	Gansu, Sunanyuguzizhixian	CVH	Confirmed
	99.4108	33.4826	HNWP152166	-	-	Qinghai, Darixian	CVH	Confirmed
	99.7113	38.3279	WUK0101066	Buqiu Zhong	1958-07-29	Qinghai, Niuxin Mountain	CVH	Confirmed
	99.7333	35.5403	WUK0290402	Tingnong He	1965-08-23	Qinghai, Hekaqieji Mountain	CVH	Confirmed
	99.7943	34.5040	HNWP172134	H. B. G	1993-08-06	Qinghai, Dawuxiang	CVH	Confirmed
	99.8609	36.9956	-	-	--	Qinghai, Qunjicun	CVH	Confirmed
N. incisum	100.0648	36.5383	19750808	Benzhao Guo	1975-08-08	Qinghai, Xinzhe	NSII	Confirmed
(*N* = 109)	100.1851	29.8952	SM715003986	Lipu's group	-	Sichuan, Lazhe	CVH	Confirmed
	100.1971	34.4904	HNWP101264	-	1981-07-06	Qinghai, Dangluoniyagemagou	CVH	Confirmed

100.2875	30.9457	SM715003987	-	-	Sichuan, Larima	CVH	Confirmed
100.4498	38.9259	20150608	Yun Jia	2015-06-08	Gansu, Zhangye	Current study	Confirmed
100.4784	32.3009	20160722	Weibing Fan	2016-07-22	Sichuan, Zudashan	Current study	Confirmed
100.5467	35.2760	19900816	Yuhuo Wu	1990-08-16	Qinghai, Maxiuxiang	NSII	Confirmed
100.5504	32.9097	HNWP74136	Benzhao Guo	1978-08-12	Qinghai, Banma	CVH	Confirmed
100.6510	28.2637	HNWP163690	Yuhuo Wu	1990-07-26	Qinghai, Longmugongma	CVH	Confirmed
100.6759	31.4921	SM715003988	-	-	Sichuan, Rendagongshe	CVH	Confirmed
100.8798	36.9606	-	-	-		NSII	Unconfirmed
100.8846	35.6981	HNWP0211785	Futun Yang	1970-09-18	Qinghai, Junmachuang	CVH	Confirmed
101.0055	33.4739	HNWP23913	Zang Yao's group	1971-07-31	Qinghai, Nianbao Mountain	CVH	Confirmed
101.0597	32.1482	SM715004004	-	-	Sichuan, Shangzhai	CVH	Confirmed
101.0998	33.3834	HNWP25387	Guoluo's group	1971-07-12	Qinghai, Suhurimagongshe	CVH	Confirmed
101.1631	36.6363	19570707	Qinghai's group	1957-07-07	Qinghai, Huangyuanxian	NSII	Confirmed
101.3272	30.0611	20160806	Weibing Fan	2016-08-06	Sichuan, Yajiang	Current study	Confirmed
101.3827	31.4693	19750928	-	1975-09-28	Sichuan, Jinchuanxian	NSII	Confirmed
101.4009	37.1007	-	-	-	Qinghai, Machangzhuangcun	NSII	Confirmed
101.4354	35.1392	HNWP18837	-	1967-08-03	Qinghai, Zekuxian	CVH	Confirmed
101.4905	37.1206	-	-	-	Qinghai, Datongxian	CVH	Confirmed
101.5445	36.5797	HNWP153392	Tingnong He	1989-07-20	Qinghai, Qunjiaxiang	CVH	Confirmed

101.5563	34.5113	HNWP18767	-	1967-07-16	Qinghai, Ningmutexiang	CVH	Confirmed
101.5596	31.8943	21387	-	-	Sichuan, Wanlinxilizhai	NSII	Confirmed
101.5861	26.9401	-	-	-	Sichuan, Panzhihua	NSII	Confirmed
101.6351	28.9178	SM715003968	-		Sichuan, Sanyanlonggongshe	NSII	Confirmed
101.6690	33.8507	20080917	-	2008-09-17	Gansu, Dianzhangou	NSII	Confirmed
101.6890	32.8584	20130821	Yun Jia	2013-08-21	Qinghai, Jiuzhixian	Current study	Confirmed
101.7313	37.4583	-	-	-	Qinghai, Menyuanxian	CVH	Confirmed
101.7524	30.9670	SM715003995	-	-	Sichuan, Dandonggongshe	CVH	Confirmed
101.7553	29.9579	SM715003971	-	-	Sichuan, Ertaizi	CVH	Confirmed
101.7679	36.6407	-	-	-	Qinghai, Xiningshi Chengbaiqu	NSII	Confirmed
101.8048	31.5275	19750928	Sichuan’s group	1975-09-28	Sichuan, Anningqu	NSII	Confirmed
101.8527	37.1496	20130809	Zhonghu Li	2013-08-09	Qinghai, Datongxian Shangtancun	Current study	Confirmed
101.8873	29.5993	19820702	Zhibei’s group	1982-07-02	Sichuan, Ludingxian	NSII	Confirmed
101.9669	29.9889	SM715003985	Zi Kang	-	Sichuan, Chengjiao	CVH	Confirmed
101.9692	30.0551	19530925	Shanyong Chen	1953-09-25	Sichuan, Huopao Mountain	NSII	Confirmed
102.0076	35.5228	20130816	Zhonghu Li	2013-08-16	Gansu, Maquxiancheng	Current study	Confirmed
102.0233	32.0193	SM715003955	-	-	Sichuan, Maerkang	CVH	Confirmed
102.0703	33.9992	19610804	-	1961-08-04	Gansu, Maquxian	NSII	Confirmed
102.1201	29.7477	19800907	-	1980-09-07	Sichuan, Ludingxian	NSII	Confirmed
102.1853	30.9335	SM715003959	-	-	Sichuan, Danba	CVH	Confirmed

102.2285	31.9057	SM715003965	Jiudan Li	-	Sichuan, Aba Maerkang	CVH	Confirmed
102.2979	38.1388	20160912	Weibing Fan	2016-09-12	Qinghai, Datongxian Dongxialinchang	Current study	Confirmed
102.4297	36.5078	HNWP130509	Yuhuo Wu	1986-07-22	Qinghai, Yaotailinchang	CVH	Confirmed
102.4319	36.8918	HNWP130677	Yuhuo Wu	1986-07-15	Qinghai, Beishanglinchang	CVH	Confirmed
102.4775	34.3926	-	-	-	Gansu, Larenguanxiang	NSII	Confirmed
102.5066	35.0230	13699	K.T. Fu	-	Gansu, Xiahexian	NSII	Confirmed
102.5783	30.0788	-	-	-	Sichuan, Yaan Tianquexian	CVH	Confirmed
102.6387	32.1214	SM715003917	Jiudan Li	-	Sichuan, Sandaguxiang	CVH	Confirmed
102.6411	32.7361	19640623	Bingzhi Ni	1964-06-23	Sichuan, Hongyuanxian	NSII	Confirmed
102.7612	37.2809	WUK0156621	Yeqi He		Gansu, Tianzhu	CVH	Unconfirmed
102.7759	36.1577	19580823	Yaoting Zhang	1958-08-23	Qinghai, Minghe	NSII	Confirmed
102.8176	30.3683	20150915	Yun Jia	2015-09-15	Sichuan, Yaanxian Baoxingxian	Current study	Confirmed
102.8955	33.6688	SM715003907	-	-	Sichuan, Baxigongshe	CVH	Confirmed
102.8992	30.3674	20150915	Yun Jia	2015-09-16	Sichuan, Baoxingxian	Current study	Confirmed
103.0554	32.1653	SM715003918	Jiudan Li	-	Sichuan, Aba Sandaguxiang	CVH	Confirmed
103.2527	36.6169	-	-	-	Gansu, Yongdengxian	NSII	Confirmed
103.2543	37.9120	20160727	Weibing Fan	2016-07-27	Gansu, Tianzhuxian	Current study	Confirmed
103.2943	31.1687	SM715003979	Tianlun Dai	-	Sichuan, Wenchuan	CVH	Confirmed
103.3875	32.7876	19840802	Fading Bo	1984-08-02	Sichuan, Huangsheng	NSII	Confirmed

103.3936	34.6144	14857	W.Y. H	-	Gansu, Zhuonixain	CVH	Confirmed
103.4009	29.5070	19640705	Qiqing Li	1964-07-05	Sichuan, Emei Mountain	CVH	Confirmed
103.5327	32.6254	19840830	Jin Yao	1984-08-30	Sichuan, Songpanbu	CVH	Confirmed
103.5704	34.0056	19600923	Zexiang Pen	1960-09-23	Gansu, Diebuxian	CVH	Confirmed
103.6290	35.2580	19920709	Ze Yang	1992-07-09	Gansu, Lianhua Mountain	CVH	Confirmed
103.6319	34.7426	WUK0092068	W.Y. H	1940-09-22	Gansu, Lintan	CVH	Confirmed
103.6350	31.8557	SM715003973	Maowen’s group	-	Sichuan, Maowenqiangzuzizhixian	CVH	Confirmed
104.0198	31.2936	-	-	-	Sichuan, Xuemensi	CVH	Confirmed
104.0679	30.6799	19790409	-	1979-04-09	Sichuan, Chengdou	NSII	Confirmed
104.2467	34.4296	WUK0033853	T.P. Wang	1937-08-23	Gansu, Minxian Min Mountain	CVH	Confirmed
104.2583	31.9625	-	-	-	Sichuan, Mianyang Beichuan	NSII	Confirmed
104.3263	33.6348	19980721	Bailongjiang’s group	1998-07-21	Gansu, Zhouquxian	NSII	Confirmed
104.3608	35.7666	19590907	-	1959-09-07	Gansu, Yuzhong Lapulen	NSII	Confirmed
104.4043	32.4469	-	-	-	Sichuan, Mianyang Pinwuxian	CVH	Confirmed
104.5106	34.1207	20160802	Weibing Fan	2016-08-02	Gansu, Zhouqu Lizigou	Current study	Confirmed
104.7842	32.9472	19590801	Wenxian’s group	1959-08-01	Gansu, Wenxian Zhongzhai	NSII	Confirmed
107.4168	33.9429	21387	Zhiping Wei	-	Shaanxi, Taibai	CVH	Confirmed
107.7010	34.0535	19561008	Qunjun Fu	1956-10-08	Shaanxi, Taibai Mountain	CVH	Confirmed
108.2255	34.9387	19380908	W.Y. H	1938-09-08	Shanxi, Meixian	NSII	Confirmed
108.7797	33.8532	21043	Kunjun Fu	-	Shanxi, Jiwozi	NSII	Confirmed

109.4567	31.9392	-	-	-	Shaanxi, Ankang	NSII	Confirmed
99.5636	35.5586	HNWP001501	-	1963-07-15	Qinghai, Tangxia	NSII	Confirmed
99.6155	38.8369	WUK0157576	Yeqi He	1959-06-30	Gansu, Qilian Mouantain	CVH	Confirmed
99.6734	34.5688	HNWP161380	Yuhuo Wu	1990-08-07	Qinghai, Maqin	CVH	Confirmed
99.9926	31.6229	SM715004007	-	-	Sichuan, Ganzi	CVH	Confirmed
100.2954	34.4774	HNWP104595	Weiyi Wang	1983-07-29	Qinghai, Makehe	CVH	Confirmed
100.5401	32.8929	NAS00022707	Dashang Luo	1980-08-06	Qinghai, Banmaxian Banqi	CVH	Confirmed
100.6181	35.1492	HNWP171412	H. B. G	1993-07-22	Qinghai, Tongde	CVH	Confirmed
100.9729	36.9433	WUK0459457	Huangtu's group	1985-07-16	Qinghai, Hebeizangzu Menyuan	CVH	Confirmed
101.0885	38.7846	WUK0155928	Yeqi He	1959-07-09	Gansu, Shandan Dahuang Mountain	CVH	Confirmed
101.1252	30.9795	-	-	-	Sichuan, Daofu Wariqu	CVH	Confirmed
101.2070	36.6390	HNWP133556	Jinye Ding	1961-09-12	Qinghai, Huangyuanxian	CVH	Confirmed
101.2794	27.9286	-	-	-	Sichuan, Mulizangzu	CVH	Confirmed
101.3203	31.8562	20160720	Weibing Fan	2016-07-20	Sichuan, Mazixiang	Current study	Confirmed
101.3888	35.1684	HNWP22991	Lihua Zhou	1970-08-25	Qinghai, Zekuxian	CVH	Confirmed
101.4008	37.0840	20140809	Weibing Fan	2014-08-09	Qinghai, Datongxian Qinlinxiang	Current study	Confirmed
101.4667	38.7167	20150607	Yun Jia	2015-06-07	Gansu, Zhangye	Current study	Confirmed
101.4771	35.9343	HNWP152898	Shangwu Liu	1989-07-24	Qingahi, Guidexian	CVH	Confirmed

	101.5159	34.5667	HNWP31962	Benzhao Guo	1972-07-19	Qinghai. Henanmenguzu	CVH	Confirmed
	101.6157	36.4716	HNWP153353	Tingnong He	1989-07-20	Qinghai, Huangzhongxian Qunjia	CVH	Confirmed
	101.6856	36.9269	HNWP154640	Lijuan Mei	1989-08-11	Qinghai, Datongxain	CVH	Confirmed
	101.7066	32.9024	-	-	-	Sichuan, Abaxian	CVH	Confirmed
	101.7608	37.5031	HNWP186601	Zhenlan Wu	1999-08-25	Qinghai, Menyuan	CVH	Unconfirmed
	101.7846	36.6306	WUK0011086	H. Ho	1930-08-03	Qinghai, Xining	CVH	Confirmed
N. franchetii	101.8527	37.1496	20130809	Zhonghu Li	2013-08-09	Qianghai, Datongxian Shangtancun	Current study	Confirmed
(*N* = 45)	101.8657	30.1413	-	-	-	Sichuan, Yidun	CVH	Confirmed
	101.9781	36.3938	HNWP174075	Xuefeng Lu	1994-08-28	Qinghai, Pingan	CVH	Confirmed
	102.0273	35.5399	HNWP102607	Benzhao Guo	1982-08-17	Qinghai, Huangnanzangzu	CVH	Confirmed
	102.0273	35.5399	HNWP48288	Zhibing Wang	1975-08-17	Qinghai, Maixiulinchang	CVH	Confirmed
	102.0703	33.9992	20150903	Yun Jia	2015-09-03	Gansu, Maquxian	Current study	Confirmed
	102.1521	36.8521	HNWP48251	Benzhao Guo	1975-09-13	Qinghai, Huzhutuzuzizhixian	CVH	Confirmed
	102.2000	30.5666	20160719	Weibing Fan	2016-07-19	Sichuan, Danbaxian Dongguxiang	Current study	Confirmed
	102.2224	31.8991	-	Shanyong Chen	-	Sichuan, Abazangzuzizhizhou	CVH	Confirmed
	102.3505	37.1864	20130816	Zhonghu Li	2013-08-16	Gansu, Maqu	Current study	Confirmed
	102.4382	36.5098	HNWP001502	Qingan’s group	1959-09-12	Qinghai, Leduxian	CVH	Confirmed
	102.4751	35.6778	HNWP27549	Benzhao Guo	1971-09-20	Qinghai, Mengda	CVH	Confirmed
	102.5217	35.2025	WUK0033935	K.T. Fu	1937-07-01	Gansu, Xiahe Qingshui	CVH	Confirmed

102.7054	36.0847	20130811	Zhonghu Li	2013-08-11	Qinghai, Minhexian	Current study	Confirmed
102.7963	36.3270	WUK0100345	Mingfang Zhong	1958-10-09	Qinghai, Qilisi	CVH	Confirmed
102.8176	30.3683	20150915	Yun Jia	2015-09-15	Sichuan, Yaan Baoxing	Current study	Confirmed
102.8741	35.7181	-	-	-	Gansu, Linxaizhou Jishishan	Current study	Confirmed
102.8746	36.7248	20160911	Weibing Fan	2016-09-11	Qinghai, Qinanxian Qunsilinchuang	Current study	Confirmed
102.9618	33.5758	20130817	Zhonghu Li	2013-08-17	Sichuan, Ruoergaixian	Current study	Confirmed
103.0212	31.5795	-	-	-	Sichuan, Abazhou Lixian	NSII	Confirmed
103.1321	28.3286	-	-	-	Sichuan, Meiguxian	CVH	Confirmed
103.2608	36.7361	HNWP165839	Tingnong He	1991-07-10	Gansu, Yongdengxian	CVH	Confirmed
103.2670	31.0840	-	-	-	Sichuan, Wolong	CVH	Confirmed
103.3487	35.4249	-	-	-	Gansu, Linxiazhou Hezhengxian	Current study	Confirmed
103.4026	37.5991	20160727	Zhonghu Li	2016-07-27	Gansu, Wuweitianzhutan Mountain	Current study	Confirmed
103.5071	34.5895	WUK0067161	T.P. Wang	1936-07-24	Gansu, Zhuoni	CVH	Confirmed
103.5989	32.6379	-	-	-	Sichuan, Huangtengge	CVH	Confirmed
103.7572	36.0758	WUK0079689	Mingfang Wang	1941-07-03	Gansu, Xinglongshan	CVH	Confirmed
103.85348	31.6811	SM715003883	Maowen’s group	-	Sichuan, Maoxian Songping	CVH	Confirmed
103.9551	36.0039	20130812	Zhonghu Li	2013-08-12	Gansu, Hepinzhen Guantangou	Current study	Confirmed
103.9837	35.1236	20160731	Zhonghu Li	2016-07-31	Gansu, Weiyuanxian Huiyuanzhen	Current study	Confirmed
104.1125	35.8431	WUK0034009	K.T. Fu	1937-07-17	Gansu, Yuzhong Lapulen	CVH	Confirmed
104.2480	34.2263	20151106	Zhonghu Li	2015-11-06	Gansu, Longnan Niujiacun	Current study	Confirmed

	104.6744	35.3104	20130805	Zhonghu Li	2013-08-05	Gansu, Yuzhong Mapoyangzhai	Current study	Confirmed
	105.0309	33.6442	20160804	Weibing Fan	2016-05-04	Gansu, Zhouquxian Gongbaxiang	Current study	Confirmed
	105.7260	34.5686	WUK0162035	Kubjun Fu	1960-08-08	Gansu, Tianshui	CVH	Confirmed
	111.5310	37.8906	20150903	Zhonghu Li	2015-09-03	Shanxi, Jiaocheng	Current study	Confirmed
N. oviforme ($N = 15$)	107.7011	34.0535	20140819	Weibing Fan	2014-08-19	Shaanxi, Taibai Mountain	Current study	Confirmed
	106.5856	35.1610	20150824	Zhonghu Li	2015-08-24	Gansu, Xihua Niushezhen	Current study	Confirmed
	106.6734	35.0690	20150822	Weibing Fan	2015-08-22	Shaanxi, Longxian Tiemahe	Current study	Confirmed
	108.5268	33.9248	20150704	Weibing Fan	2015-07-04	Shaanxi, Zhuque	Current study	Confirmed
	108.8230	33.8205	20150519	Zhonghu Li	2015-05-19	Shaanxi, Jiwozi	Current study	Confirmed
	105.1848	34.5744	20150717	Yun Jia	2015-07-17	Gansu, Gangguxian	Current study	Confirmed
	106.5821	35.1609	20141001	Weibing Fan	2014-10-01	Gansu, Xihuazhen Caotancun	Current study	Confirmed
	108.7230	33.7205	20160731	Zhonghu Li	2016-07-31	Shaanxi, Fenyukou	Current study	Confirmed
	108.2716	33.2794	20160804	Zhonghu Li	2016-08-04	Shaanxi, Pinheliang	Current study	Confirmed
	107.1761	35.5032	20160726	Yun Jia	2016-07-26	Shaanxi, Guanshan	Current study	Confirmed
	108.6230	33.6205	201678	Yun Jia	2016-07-08	Shaanxi, Fenyukou	Current study	Confirmed
	108.8230	33.8205	20160708	Zhonghu Li	2016-07-08	Shaanxi, Changanqu	Current study	Confirmed
	106.4023	35.1588	20160812	Zhonghu Li	2016-08-12	Gansu, Huaxiangxian	Current study	Confirmed
	106.6531	35.2182	20160817	Yun Jia	2016-08-17	Gansu, Guanshang	Current study	Confirmed
	108.5268	33.9248	20160822	Yun Jia	2016-08-22	Shaanxi, Huxian	Current study	Confirmed

Table S6 Gene diversity, nucleotide diversity, and haplotype frequencies of the cpDNA and mtDNA sequences for all studied populations of four *Notopterygium* species. *N*, number of samples; θ_{wt} , Watterson's θ; π_t, total nucleotide diversity. Note: H(a) b means that haplotype "a" is shown by "b" individuals. SD (standard deviation).

Species	Population	cpDNA				mtDNA			
		θ_{wt}	π_t (SD) × 10^{-2}	cpDNA haplotypes	*N*	θ_{wt}	π_t (SD) × 10^{-2}	mtDNA haplotypes	*N*
	A	0	0	H(1) 1 H(2) 5	6	0	0	H(1) 6	6
	B	0	0	H(2) 3	3	0	0	H(1) 2	2
	C	0	0	H(2) 5	5	0	0	H(1) 6	6
	D	0.4	0.023 (0.013)	H(3) 4 H(4) 1	5	0	0	H(2) 6	6
	E	0	0	H(5) 5	5	0	0	H(2) 5	5
	G	0.5	0.084 (0.045)	H(6) 3 H(7) 1	4	0.7	0.238 (0.106)	H(1) 3 H(3) 1	4
	H	_	_	H(8) 1	1	0	0	H(1) 6	6
	I	0	0	H(2) 5	5	0	0	H(1) 6	6
	J	1	0.113 (0.040)	H(5) 1 H(7) 1 H(9) 1	3	0	0	H(1) 4	4
	L	0	0	H(5) 2	2	0	0	H(2) 5	
	N	1	0.057 (0.028)	H(10) 1 H(11) 1	2	0.6	0.079 (0.023)	H(1) 2 H(2) 3	5
	Q	1	0.112 (0.056)	H(5) 1 H(12) 1	2	0.4	0.053 (0.031)	H(1) 1 H(2) 4	5
	S	0	0	H(13) 2	2	0	0	H(1) 2	2
N. incisum	T	0	0	H(5) 2	2	0		H(2) 2	2
	U	0	0	H(8) 1 H(14) 1	2	0.6	0.079 (0.023)	H(1) 3 H(2) 2	5
	V	0.4	0.045 (0.027)	H(8) 4 H(15) 1	5	0	0	H(1) 2	2

W	0	0	H(5) 1 H(6) 3 H(16) 1	5	0.4	0.053 (0.031)	H(1) 4 H(2) 1	5
X	0	0	H(5) 1 H(16) 1	2	0	0	H(1) 2	2
Y	0	0	H(8) 2	2	0	0	H(1) 2	2
Z	0	0	H(5) 1 H(17) 1	2	0	0	H(1) 1 H(4) 1	2
HA	0.4	0.023 (0.013)	H(2) 4 H(18) 1	5	0	0	H(1) 2	2
HB	0.6	0.102 (0.030)	H(19) 1 H(20) 2 H(21) 2	5	0	0	H(1) 2	2
HC	1	0.112 (0.056)	H(19) 1 H(22) 1	2	0	0	H(1) 2	2
HF	0	0	H(3) 2	2	0	0	H(2) 5	5
HH	0	0	H(8) 2	2	0	0	H(1) 2	2
HI	0	0	H(2) 5	5	0	0	H(1) 2	2
KA	0	0	H(23) 3	3	0	0	H(1) 6	6
KC	0	0	H(23) 2 H(24) 1 H(25) 1	4	0	0	H(1) 5	5
KD	0	0	H(24) 5	5	0.333	0.044 (0.028)	H(1) 5 H(5) 1	6
KF	0	0	H(24) 5	5	0	0	H(1) 6	6
KG	0.4	0.067 (0.040)	H(26) 4 H(27) 1	5	0	0	H(1) 2	2
KH	0	0	H(24) 5	5	0	0	H(5) 6	6
KI	0	0	H(23) 3	3	0	0	H(1) 5	5
KJ	0	0	H(23) 6	6	0	0	H(1) 6	6
KK	_	_	H(23) 1	1	0	0	H(1) 6	6
KL	0	0	H(24) 2	2	0	0	H(5) 5	5
KM	0	0	H(28) 5	5	0	0	H(1) 2	2
KO	1	0.056 (0.028)	H(23) 1 H(29) 1	2	0	0	H(1) 2	2
KQ	0.667	0.037 (0.018)	H(24) 1 H(27) 1 H(30) 1	3	0	0	H(1) 4	4

N. franchetii	KR	0	0	H(31) 2	2	0	0	H(1) 2	2
	KV	0	0	H(24) 2	2	0	0	H(1) 3	3
	KX	0	0	H(24) 5 H(32) 1	6	0	0	H(5) 6	6
	KZ	0.6	0.034 (0.007)	H(24) 3 H(33) 3	6	0	0	H(1) 6	6
	YA	0.6	0.010 (0.030)	H(23) 3 H(34) 2	5	0	0	H(1) 2	2
	YB	0	0	H(24) 2	2	0	0	H(1) 2	2
	YC	0	0	H(35) 5	5	0	0	H(1) 2	2
	YD	0	0	H(23) 2	2	0	0	H(1) 2	2
	YE	0	0	H(23) 2	2	0	0	H(1) 2	2
	YF	0.7	0.067 (0.027)	H(35) 2 H(36) 1 H(37) 1 H(38) 1	5	0	0	H(1) 2	2
	YH	_	_	_		0	0	H(1) 2	2
	YJ	_	_	_		0	0	H(1) 2	2
	YK	0	0	H(24) 2	2	0	0	H(5) 5	5
	YM	0	0	H(23) 1 H(24) 1	2	0	0	H(1) 2	2
	LA	0	0	H(39) 5	5	0	0	H(5) 5	5
	LB	0	0	H(40) 2	2	0	0	H(5) 4	4
	LC	0	0	H(41) 2	2	0	0	H(5) 5	5
	LD	0.5	0.056 (0.030)	H(42) 1 H(43) 3	4	0	0	H(1) 4	4
	LE	0.5	0.056 (0.030)	H(44) 3 H(45) 1	4	0	0	H(1) 4	4
	LF	0.6	0.034 (0.010)	H(27) 3 H(46) 2	5	0	0	H(1) 2	2
N. oviforme	LG	0	0	H(47) 2	2	0	0	H(5) 5	5
	LK	0.667	0.187 (0.088)	H(48) 2 H(49) 1	3	0	0	H(1) 2	2

	LO	0	0	H(50) 2	2	0	0	H(1) 2	2
	LP	_	_	H(51) 1	1	0.5	0.066 (0.035)	H(1) 1 H(5) 3	4
	LQ	0	0	H(45) 2	2	0	0	H(1) 2	2
	LR	_	_	_		0	0	H(1) 2	2
	LT	0.4	0.067 (0.040)	H(52) 4 H(53) 1	5	0	0	H(5) 5	5
	LV	_	_	_		0	0	H(1) 4	4
	LU	0	0	H(54)2	2	0	0	H(5) 5	5
	LQA	0	0	H(55) 1 H(56) 1	2	0	0	H(1) 2	2
	LQB	0	0	H(57) 2	2	0	0	H(1) 2	2
N. forrestii	LQC	0	0	H(56) 2	2	0	0	H(1) 2	2
	LQD	0	0	H(56) 4	4	0	0	H(1) 2	2

Table S7 Analysis of molecular variance (AMOVA) based on variation at 10 nuclear loci, cpDNA, and mtDNA in four species of *Notopterygium*.

Source of variation	d. f.	SS	VC	Variation (%)	Fixation index
**Nuclear locus 35**					
Among all species	3	109.603	0.34136	66.30853	F_{CT} = 0.66309***
Among populations within species	68	35.168	0.06192	12.02688	F_{ST} = 0.78335***
Within populations	404	45.058	0.11153	21.66459	F_{SC} = 0.35697***
Total	475	189.830	0.51481		
Among populations within *N. incisum*	25	0.618	0.00108	5.55940	F_{ST} = 0.05559
Within populations	128	2.350	0.01836	94.44060	
Total	153	2.968	0.01944		
Among populations within *N. franchetii*	26	19.714	0.08481	44.91591	F_{ST} = 0.44916***
Within populations	183	19.033	0.10401	55.08409	
Total	209	38.748	0.18882		
Among populations within *N. oviforme*	14	10.211	0.08668	27.50894	F_{ST} = 0.27509***
Within populations	73	16.675	0.22842	72.49106	
Total	87	26.886	0.31511		

Among populations within *N. forrestii*	3	4.625	0.20625	37.07865	F_{ST} = 0.37079***
Within populations	20	7	0.35000	62.92135	
Total	23	11.625	0.55625		
**Nuclear locus 32125**					
Among all species	3	893.262	3.08733	84.13877	F_{CT} = 0.84139***
Among populations within species	68	113.958	0.20884	5.69160	F_{ST} = 0.89830***
Within populations	380	141.800	0.37316	10.16963	F_{SC} = 0.35884***
Total	451	1149.020	3.66934		
Among populations within *N. incisum*	25	94.691	0.36862	43.73685	F_{ST} = 0.43737***
Within populations	208	98.633	0.47420	56.26315	
Total	233	193.325	0.84282		
Among populations within *N. franchetii*	26	10.913	0.06521	30.91913	F_{ST} = 0.30919***
Within populations	87	12.675	0.14569	69.08087	
Total	113	23.588	0.21090		
Among populations within *N. oviforme*	14	3.357	0.01839	11.05487	F_{ST} = 0.11055*
Within populations	61	9.025	0.14795	88.94513	
Total	75	12.382	0.16634		

Among populations within *N. forrestii*	3	4.998	0.11408	11.31197	F_{ST} = 0.11312*
Within populations	24	21.467	0.89444	88.68803	
Total	27	26.464	1.00853		
**Nuclear locus 51964**					
Among all species	3	1023.825	4.33780	89.05501	F_{CT} = 0.89055***
Among populations within species	67	98.982	0.23542	4.83308	F_{ST} = 0.93888***
Within populations	287	85.442	0.29771	6.11191	F_{SC} = 0.44158***
Total	357	1208.249	4.87092		
Among populations within *N. incisum*	25	9.651	0.03415	12.70237	F_{ST} = 0.12702***
Within populations	90	21.125	0.23472	87.29763	
Total	115	30.776	0.26888		
Among populations within *N. franchetii*	26	77.611	0.44155	54.96861	F_{ST} = 0.54969***
Within populations	135	48.833	0.36173	45.03139	
Total	151	126.444	0.80328		
Among populations within *N. oviforme*	13	11.720	0.13152	29.81107	F_{ST} = 0.29811***
Within populations	50	15.483	0.30967	70.18893	

Total	63	27.203	0.44119		
**Nuclear locus OG29988**					
Among all species	3	284.425	1.00475	88.89392	F_{CT} = 0.88894***
Among populations within species	68	26.403	0.05242	4.63755	F_{ST} = 0.93531***
Within populations	362	26.467	0.07311	6.46853	F_{SC} = 0.41757***
Total	433	337.295	1.13028		
Among populations within *N. incisum*	25	14.267	0.07289	37.67329	F_{ST} = 0.37673***
Within populations	136	16.4	0.12059	62.32671	
Total	161	30.667	0.19348		
Among populations within *N. franchetii*	26	3.394	0.01314	24.41672	F_{ST} = 0.24417***
Within populations	159	6.467	0.04067	75.58328	
Total	185	9.860	0.05381		
Among populations within *N. oviforme*	14	8.743	0.12099	64.89378	F_{ST} = 0.64894***
Within populations	55	3.600	0.06545	35.10622	
Total	69	12.343	0.18645		
**Nuclear locus 9122**					
Among all species	3	1292.616	5.59195	83.46143	F_{CT} = 0.83461***

Among populations within species	64	220.782	0.51513	7.68840	F_{ST} = 0.91150***
Within populations	308	182.633	0.59297	8.85017	F_{SC} = 0.46488***
Total	375	1696.032	6.70005		
Among populations within *N. incisum*	25	70.813	0.44976	42.90941	F_{ST} = 0.42909***
Within populations	104	62.233	0.59840	57.09059	
Total	129	133.046	1.04815		
Among populations within *N. franchetii*	25	66.561	0.28954	34.25156	F_{ST} = 0.34252***
Within populations	164	91.150	0.55579	65.74844	
Total	189	157.711	0.84533		
Among populations within *N. oviforme*	11	75.909	1.66244	66.74946	F_{ST} = 0.66749***
Within populations	32	26.500	0.82812	33.25054	
Total	43	102.409	2.49056		
Among populations within *N. forrestii*	3	7.500	0.74639	68.46748	F_{ST} = 0.68467***
Within populations	8	2.75	0.34375	31.53252	
Total	11	10.25	1.09014		
**Nuclear locus 25629**					
Among all species	3	970.297	2.58744	65.38840	F_{CT} = 0.65388***

Among populations within species	67	371.640	0.61985	15.66459	F_{ST} = 0.81053***
Within populations	481	360.625	0.74974	18.94701	F_{SC} = 0.45258***
Total	551	1702.562	3.95704		
Among populations within *N. incisum*	24	186.628	0.87198	58.40021	F_{ST} = 0.58400***
Within populations	181	112.425	0.62113	41.59979	
Total	205	299.053	1.49311		
Among populations within *N. franchetii*	26	137.728	0.53292	36.30638	F_{ST} = 0.36306***
Within populations	195	182.308	0.93491	63.69362	
Total	221	320.036	1.46783		
Among populations within *N. oviforme*	14	45.117	0.41992	40.52197	F_{ST} = 0.40522***
Within populations	79	48.692	0.61635	59.47803	
Total	93	93.809	1.03627		
Among populations within *N. forrestii*	3	2.167	0.00833	1.24287	F_{ST} = 0.01243
Within populations	26	17.200	0.66154	98.75713	
Total	29	19.367	0.66986		
**Nuclear locus 25679**					
Among all species	3	469.580	1.52596	63.59405	F_{CT} = 0.63594***

Among populations within species	66	161.439	0.29230	12.18159	F_{ST} = 0.75776***
Within populations	380	220.883	0.58127	24.22435	F_{SC} = 0.33460***
Total	449	851.902	2.39954		
Among populations within *N. incisum*	25	68.111	0.30768	31.18722	F_{ST} = 0.31187***
Within populations	148	100.475	0.67889	68.81278	
Total	173	168.586	0.98657		
Among populations within *N. franchetii*	26	27.130	0.11748	26.96786	F_{ST} = 0.26968***
Within populations	141	44.858	0.31814	73.03214	
Total	167	71.988	0.43562		
Among populations within *N. oviforme*	12	59.547	0.64192	45.38783	F_{ST} = 0.45388***
Within populations	73	56.383	0.77237	54.61217	
Total	85	115.930	1.41429		
Among populations within *N. forrestii*	3	6.652	0.21607	16.86861	F_{ST} = 0.16869**
Within populations	18	19.167	1.06481	83.13139	
Total	21	25.818	1.28088		
**Nuclear locus 29206**					
Among all species	3	3656.184	42.91421	94.17464	F_{CT} = 0.94175***

Among populations within species	36	270.892	1.28316	2.81588	F_{ST} = 0.96991***
Within populations	152	208.450	1.37138	3.00948	F_{SC} = 0.48338***
Total	191	4135.526	45.56876		
Among populations within *N. incisum*	25	82.579	0.49895	42.22879	F_{ST} = 0.42229***
Within populations	112	76.450	0.68259	57.77121	
Total	137	159.029	1.18154		
Among populations within *N. forrestii*	2	5.750	0.42115	35.37964	F_{ST} = 0.35380***
Within populations	13	10	0.76923	64.62036	
Total	15	15.750	1.19038		
**Nuclear locus OG29101**					
Among all species	3	1833.563	6.27328	90.00281	F_{CT} = 0.90003***
Among populations within species	63	155.201	0.31127	4.46581	F_{ST} = 0.94469***
Within populations	381	146.892	0.38554	5.53138	F_{SC} = 0.44671***
Total	447	2135.656	6.97009		
Among populations within *N. incisum*	24	61.627	0.30879	42.79913	F_{ST} = 0.42799***
Within populations	151	62.317	0.41269	57.20087	

Total	175	123.943	0.72148		
Among populations within *N. franchetii*	22	90.986	0.46163	49.59900	F_{ST} = 0.49599***
Within populations	161	75.525	0.46910	50.40100	
Total	183	166.511	0.93073		
Among populations within *N. oviforme*	14	2.589	0.00552	3.35918	F_{ST} = 0.03359
Within populations	57	9.050	0.15877	96.64082	
Total	71	11.639	0.16429		
**Nuclear locus OG29960**					
Among all species	3	184.057	0.71283	77.94437	F_{CT} = 0.77944***
Among populations within species	68	33.454	0.06298	6.88613	F_{ST} = 0.84830***
Within populations	332	46.058	0.13873	15.16950	F_{SC} = 0.31222***
Total	403	263.569	0.91453		
Among populations within *N. incisum*	25	7.591	0.04382	34.67918	F_{ST} = 0.34679***
Within populations	106	8.750	0.08255	65.32082	
Total	131	16.341	0.12637		
Among populations within *N. franchetii*	26	24.697	0.10265	32.78901	F_{ST} = 0.32789***
Within populations	169	35.558	0.21040	67.21099	

Total	195	60.255	0.31305		
Among populations within *N. oviforme*	14	1.167	0.01111	22.22222	F_{ST} = 0.22222
Within populations	45	1.750	0.03889	77.77778	
Total	59	2.917	0.05000		
**cpDNA**					
Among all species	3	993.201	6.44536	63.75000	F_{CT} = 0.63746***
Among populations within species	64	657.645	2.89216	28.60000	F_{ST} = 0.92350***
Within populations	157	121.433	0.77346	7.65000	F_{SC} = 0.78900***
Total	224	1772.280	10.11097		
Among populations within *N. incisum*	25	471.584	5.38424	81.74000	F_{ST} = 0.81737***
Within populations	60	72.183	1.20306	18.26000	
Total	85	543.767	6.58730		
Among populations within *N. franchetii*	24	83.578	0.84710	64.91000	F_{ST} = 0.64909***
Within populations	65	29.767	0.45795	35.09000	
Total	89	113.344	1.30505		
Among populations within *N. oviforme*	12	95.683	2.45649	77.09000	F_{ST} = 0.77088***
Within populations	26	18.983	0.73013	22.91000	

Total	38	114.667	3.18661		
**mtDNA**					
Among all species	3	19.883	0.10129	30.78693	F_{CT} = 0.30787***
Among populations within species	68	47.763	0.17964	54.59928	F_{ST} = 0.85386***
Within populations	191	9.183	0.04808	14.61378	F_{SC} = 0.78886***
Total	262	76.829	0.32901		
Among populations within *N. incisum*	25	17.869	0.16295	60.68723	F_{ST} = 0.60687***
Within populations	72	7.600	0.10556	39.31277	
Total	97	25.469	0.26850		
Among populations within *N. franchetii*	26	16.929	0.17258	93.87454	F_{ST} = 0.93875***
Within populations	74	0.833	0.01126	6.12546	
Total	100	17.762	0.18384		
Among populations within *N. oviforme*	14	12.964	0.24523	93.05841	F_{ST} = 0.93058***
Within populations	41	0.750	0.01829	6.94159	
Total	55	13.714	0.26352		

d.f., degrees of freedom; SS, sum of squares; VC, variance components; F_{CT}, differentiation among groups (i.e. the four species); F_{SC}, differentiation among populations within groups; F_{ST}, differentiation among populations. * $P < 0.05$, ** $P < 0.01$ and *** $P < 0.001$, 1000 permutations.

Table S8 Genetic differentiation at 10 nuclear loci among: (a) *Notopterygium incisum*, (b) *N. franchetii*, (c) *N. oviforme*, and (d) *N. forrestii*.

Group	a vs. b		a vs. c		a vs. d	
	F_{ST}	Da	F_{ST}	Da	F_{ST}	Da
35	0.42727***	0.00004	0.61085***	0.00090	0.97061***	0.01566
9122	0.92843***	0.02425	0.91491***	0.02155	0.88560***	0.01398
25629	0.83930***	0.01623	0.85974***	0.01471	0.86113***	0.01525
25679	0.82502***	0.01203	0.73059***	0.00851	0.65614***	0.00614
29206	0.66046***	0.00336	0.88157***	0.01374	0.91331***	0.01801
32125	0.91079***	0.01256	0.90478***	0.01234	0.90328***	0.01579
51964	0.95362***	0.01893	0.95696***	0.01774	0.96609***	0.01815
OG29101	0.95032***	0.07313	0.96442***	0.08232	0.96301***	0.08796
OG29960	0.87227***	0.00452	0.93331***	0.00415	0.92921***	0.00415
OG29988	0.95151***	0.01298	0.93928***	0.01331	0.93489***	0.01307
Average	0.83190	0.01780	0.86964	0.01893	0.89833	0.02082
**Group**	**b vs. c**		**b vs. d**		**c vs. d**	
	F_{ST}	Da	F_{ST}	Da	F_{ST}	Da
35	0.53946***	0.00062	0.94376***	0.01570	0.89922***	0.01656
9122	0.78322***	0.00561	0.89021***	0.01421	0.86320***	0.01151
25629	0.44422***	0.00096	0.78430***	0.01363	0.81260***	0.01156
25679	0.52014***	0.00134	0.86302***	0.01352	0.72129***	0.00935
29206	0.82535***	0.01423	0.91913***	0.01895	0.83515***	0.01281
32125	0.25362***	0.00002	0.88910***	0.00839	0.86860***	0.00816
51964	0.63745***	0.00085	0.91486***	0.00996	0.92282***	0.00894
OG29101	0.59045***	0.00227	0.72050***	0.00700	0.78310***	0.00429
OG29960	0.45309***	0.00032	0.45001***	0.00038	0.19547*	0.00000
OG29988	0.57380***	0.00021	0.19932***	0.00000	0.67087***	0.00023
Average	0.56208	0.00264	0.75742	0.01017	0.75723	0.00834

* $P < 0.05$, ** $P < 0.01$ and *** $P < 0.001$, 1000 permutations.

Table S9 Estimated posterior distributions of the parameters according to Approximate Bayesian Computation (ABC) for the best scenario of the demographic history for the four *Notopterygium* species.

N. incisum

**Parameter**	**N1**	**N4**	**NA**	**T1**	**T2**
Mean	8.01×10^5	9.59×10^4	4.21×10^5	8.69×10^4	1.48×10^6
**Median**	$\mathbf{8.10\times10^5}$	$\mathbf{9.09\times10^4}$	$\mathbf{4.06\times10^5}$	$\mathbf{6.40\times10^4}$	$\mathbf{1.48\times10^6}$
Mode	8.12×10^5	8.06×10^4	2.17×10^5	5.63×10^4	4.06×10^4
Lower-bound	5.55×10^5	1.21×10^4	8.08×10^4	2.36×10^4	4.68×10^4
Upper-bound	9.87×10^5	2.07×10^5	8.49×10^5	4.08×10^5	2.92×10^6

N. franchetii

**Parameter**	**N1**	**N4**	**NA**	**T1**	**T2**
Mean	6.24×10^5	1.08×10^5	3.39×10^5	8.28×10^4	1.38×10^6
**Median**	$\mathbf{6.13\times10^5}$	$\mathbf{1.00\times10^5}$	$\mathbf{3.19\times10^5}$	$\mathbf{4.73\times10^4}$	$\mathbf{1.33\times10^6}$
Mode	5.76×10^5	9.73×10^4	2.37×10^5	2.27×10^4	1.91×10^4
Lower-bound	3.49×10^5	7.69×10^3	7.23×10^4	7.55×10^3	2.44×10^4
Upper-bound	9.55×10^5	2.62×10^5	7.35×10^5	5.51×10^5	2.92×10^6

N. oviforme

**Parameter**	**N1**	**NA**	**T1**
Mean	4.53×10^5	2.48×10^5	2.79×10^5
**Median**	$\mathbf{4.25\times10^5}$	$\mathbf{2.42\times10^5}$	$\mathbf{2.14\times10^5}$
Mode	3.77×10^5	2.32×10^5	3.25×10^4
Lower-bound	2.49×10^5	1.92×10^4	1.41×10^4
Upper-bound	8.39×10^5	5.25×10^5	8.91×10^5

N. forrestii

**Parameter**	**N5**	**NA**	**T1**
Mean	1.22×10^5	4.93×10^5	3.45×10^5
**Median**	$\mathbf{1.19\times10^5}$	$\mathbf{4.71\times10^5}$	$\mathbf{3.65\times10^5}$
Mode	1.14×10^5	1.77×10^5	4.93×10^5
Lower-bound	5.78×10^4	7.65×10^4	9.53×10^4
Upper-bound	2.08×10^5	9.70×10^5	4.94×10^5

Table S10 Comparisons of the current occupied climatic niches by pairs (*A* vs. *B*) of three *Notopterygium* species, based on E-space analysis. Schoener's D_S represents niche overlap between niches (D_S; 0 = no overlap, 1 = complete overlap). The niche equivalency (eq) and niche similarity (sim) tests are considered significant ($P < 0.05$, showed in bold) when niche overlap is lower than randomly expected (niche divergence; D), or larger than randomly expected (niche conservatism; C). The niche unfilling, stability, and expansion are also presented. Not applicable cases are shown as NA.

**Distribution ranges** (comparisons *A* → *B*)		**Niche Overlap (D_S)**	**Equivalency test (*P*-value)**		**Similarity test (*P*-value)**		**Niche dynamics metrics**		
A	*B*		less eq	more eq	less sim	more sim	Unfilling	Stability	Expansion
N. franchetii	*N. incisum*	0.468	0.535	0.554	0.980	**0.040**	0.124	0.992	0.008
	N. oviforme	0.022	**0.030**	NA	0.802	0.168	0.459	0.046	0.954
N. incisum	*N. franchetii*	0.468	0.475	0.525	0.970	**0.020**	0.008	0.876	0.124
	N. oviforme	0.017	NA	NA	0.792	0.297	0.057	0.030	0.970
N. oviforme	*N. franchetii*	0.022	NA	0.950	0.901	0.158	0.954	0.541	0.459
	N. incisum	0.017	NA	NA	0.743	0.238	0.970	0.943	0.057

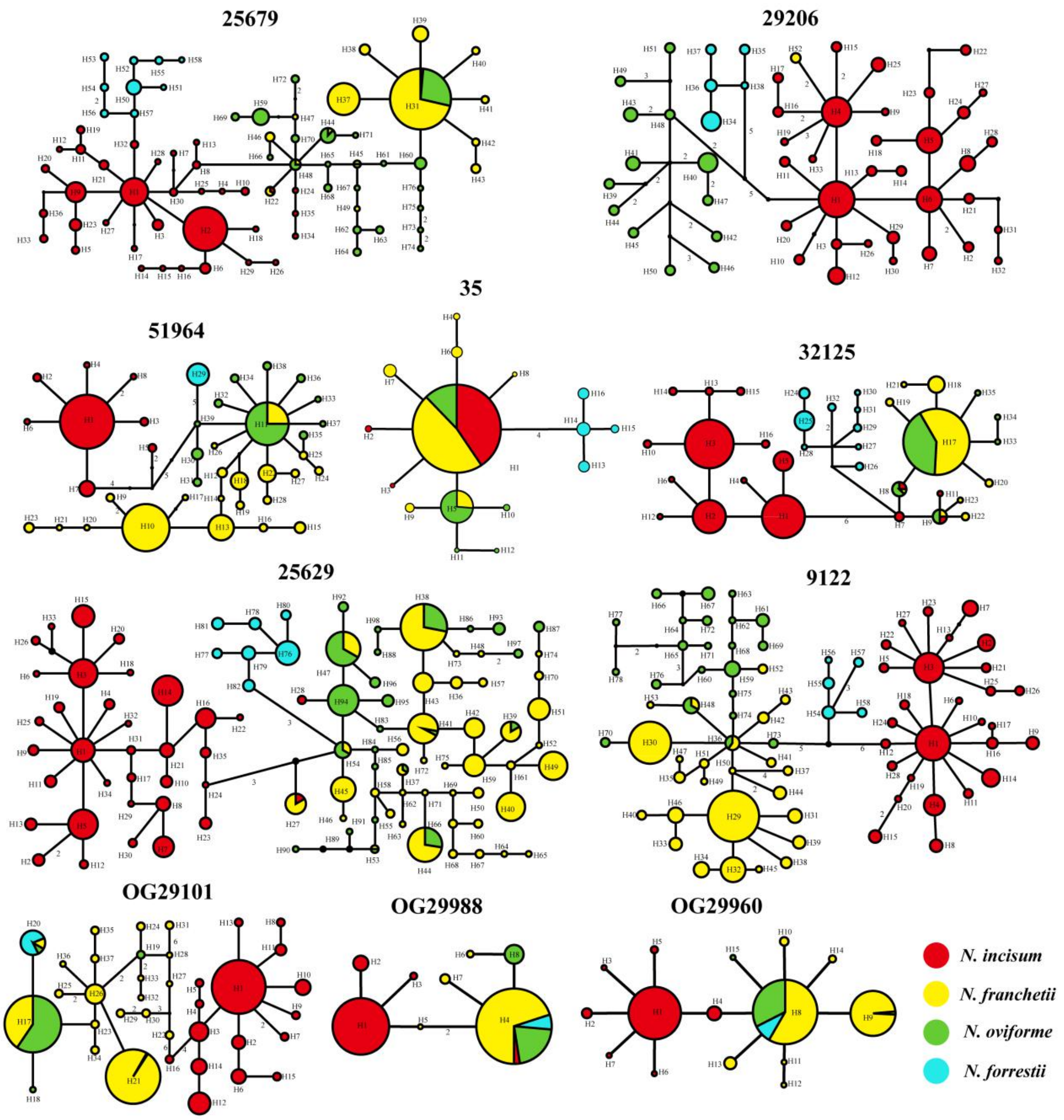

Figure S1 Haplotype (= allele) genealogies at 10 nuclear loci in the four studied *Notopterygium* species.

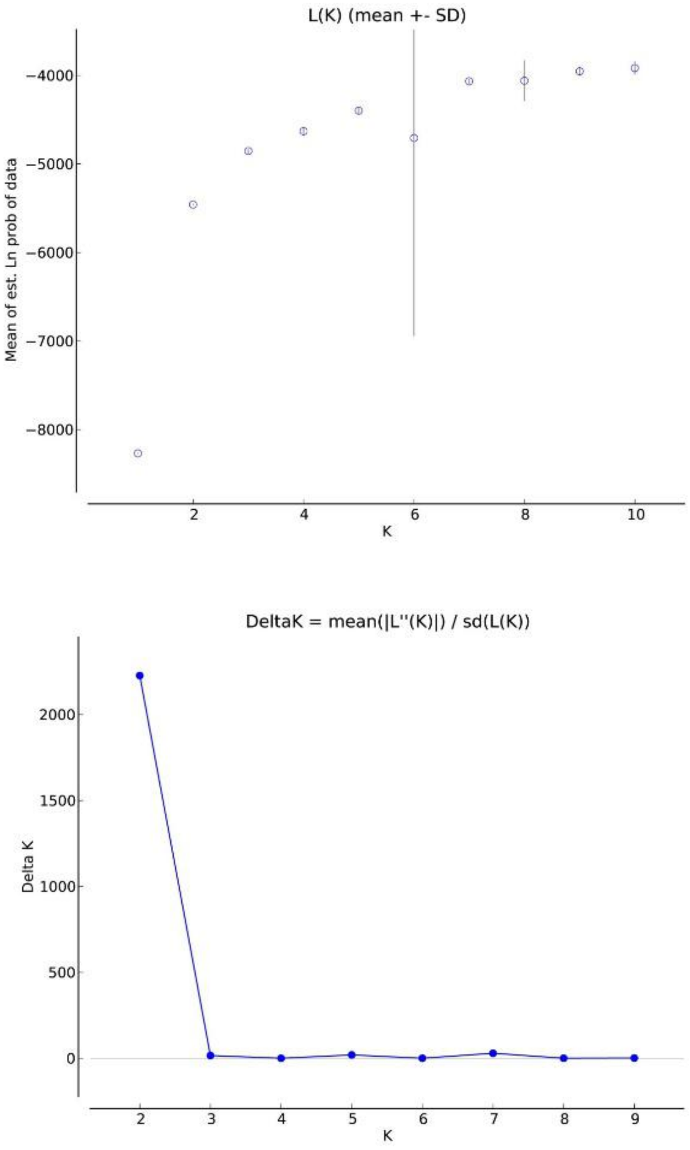

Figure S2 Two methods used to estimate the best *K* values with STRUCTURE based on nuclear markers, in the four studied *Notopterygium* species.

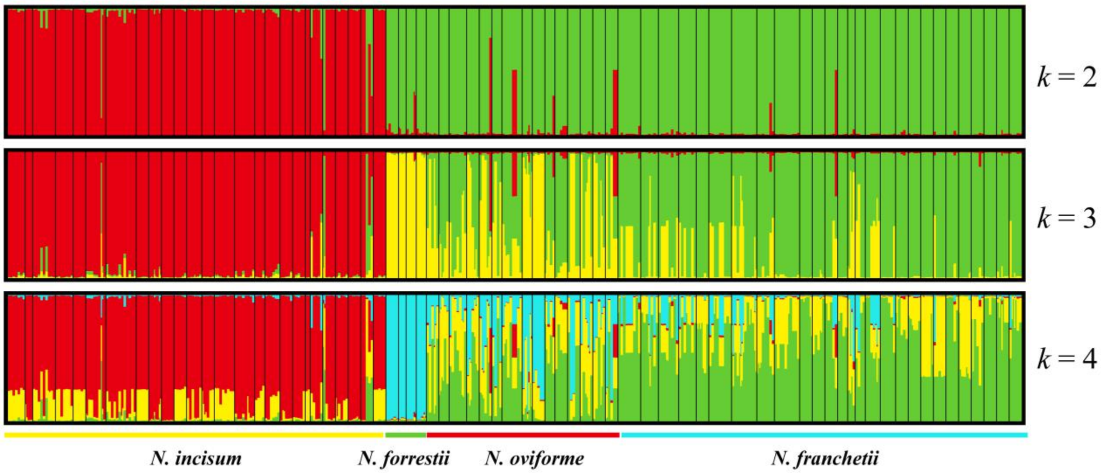

Figure S3 STRUCTURE analysis of four *Notopterygium* species when K = 2–4 clusters were assumed.

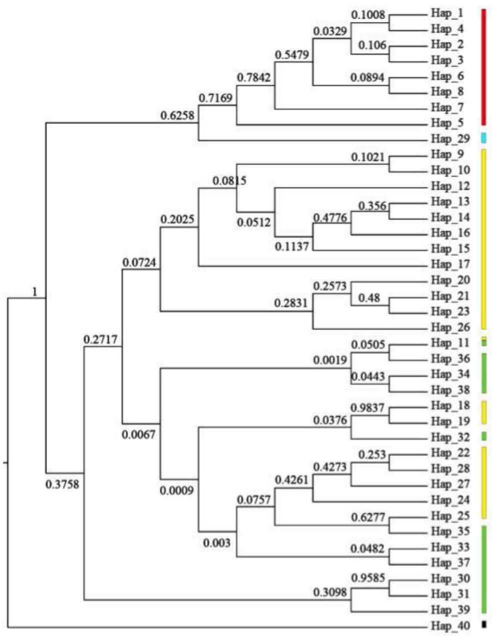

51964

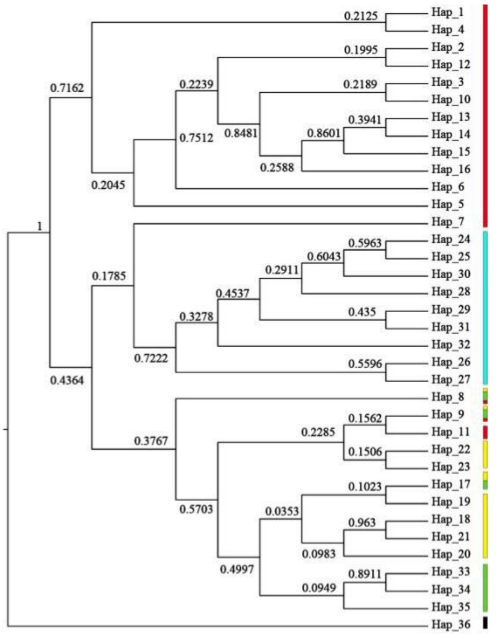

32125

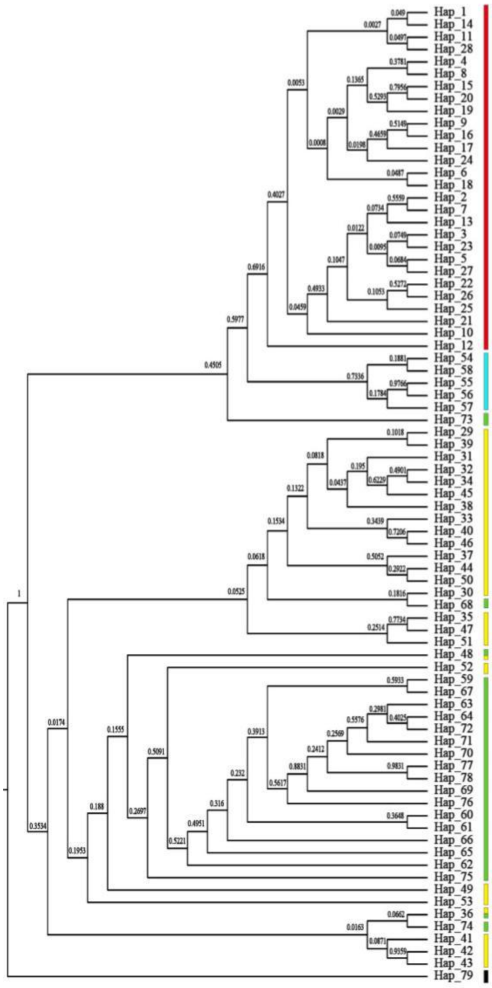

9122

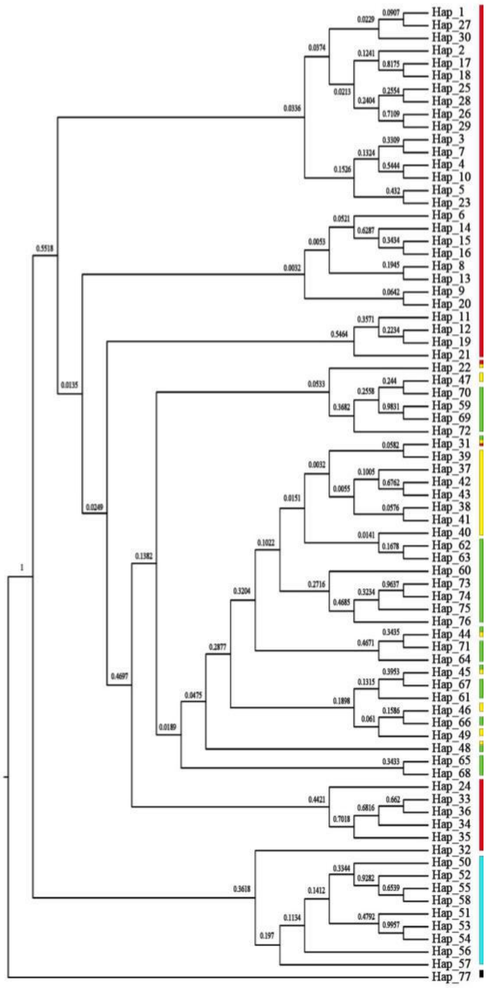

25679

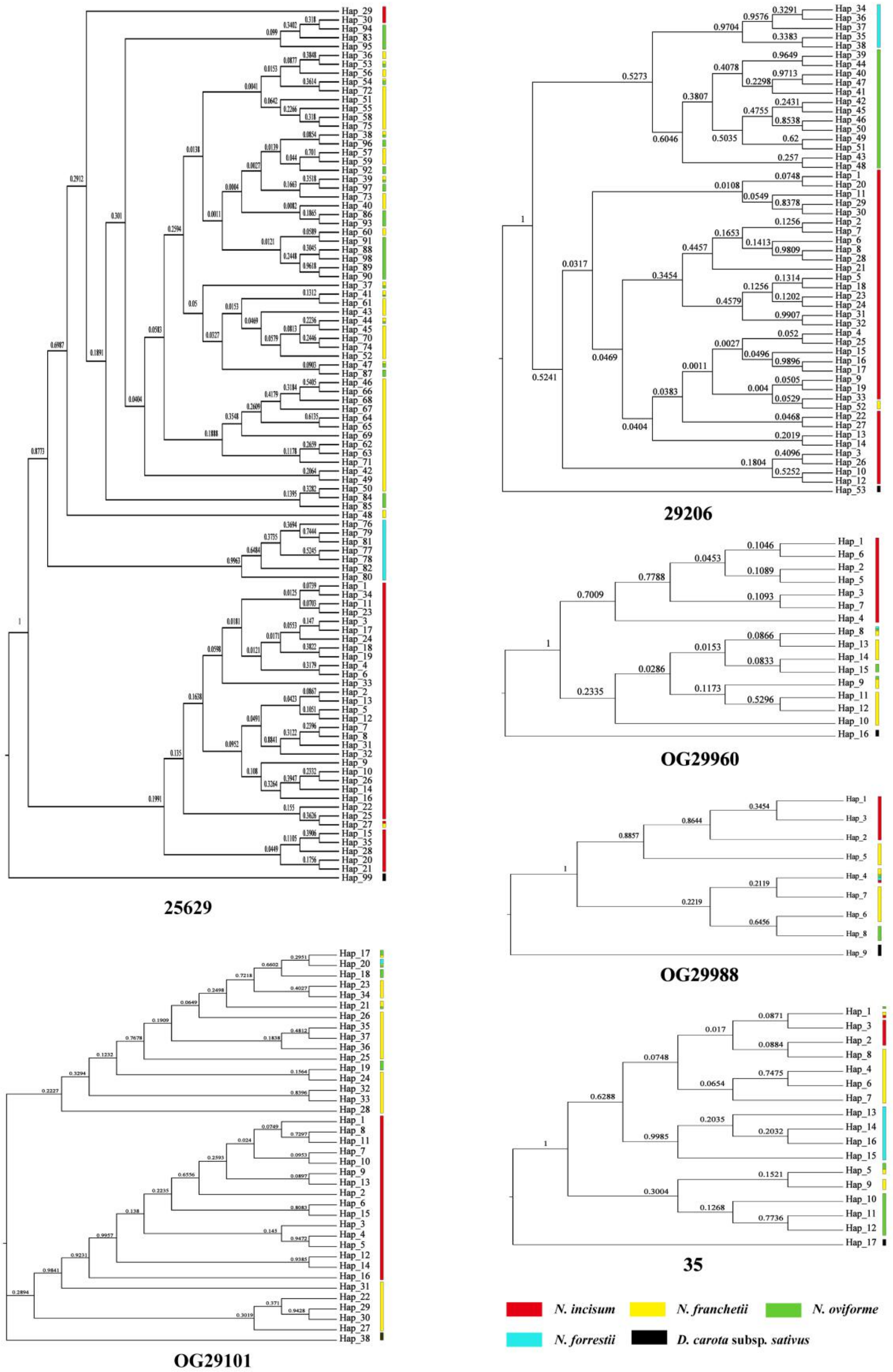

Figure S4 Bayesian inference phylogenetic trees based on each of the 10 single copy nuclear loci, in the four studied *Notopterygium* species.

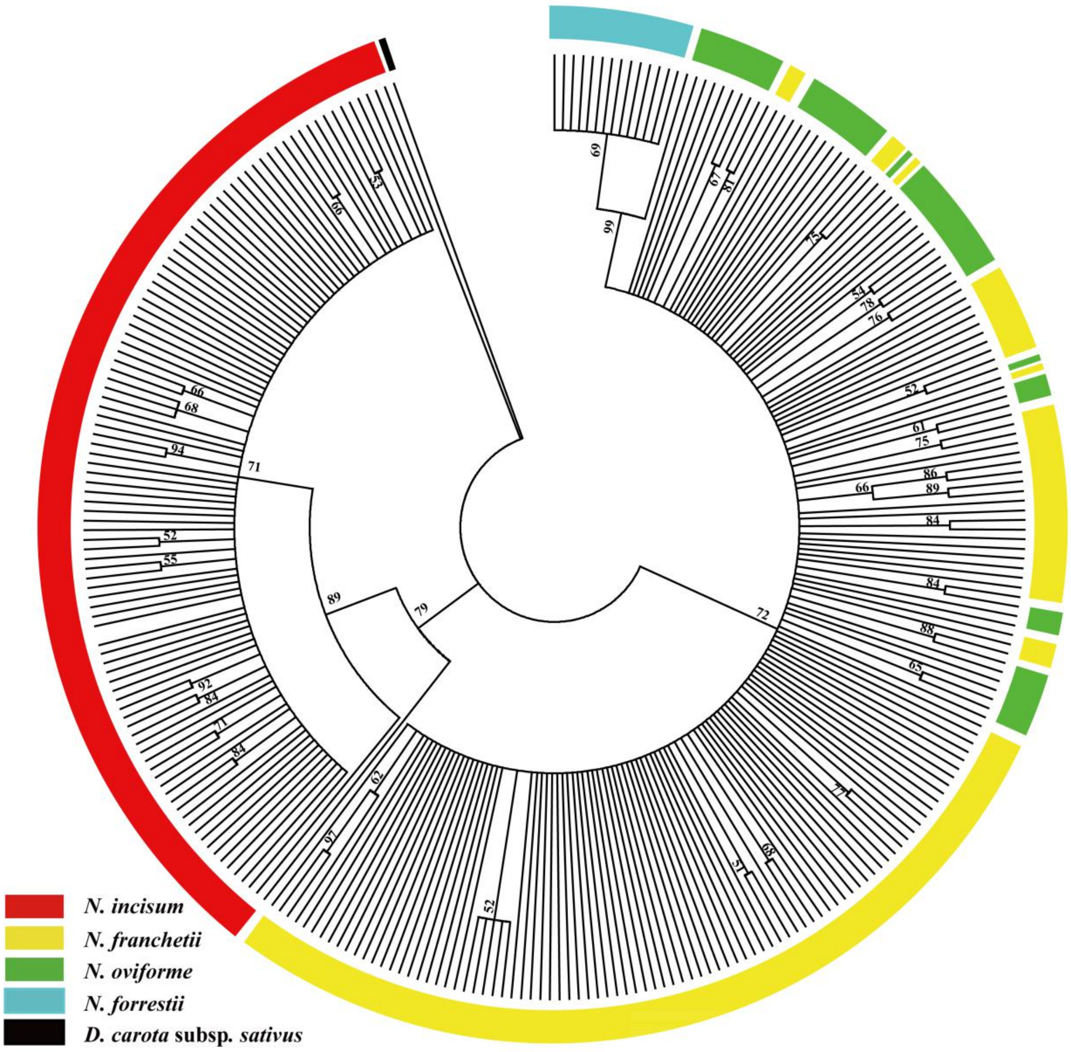

Figure S5 The Maximum Likelihood phylogenetic tree based on concatenated 10 single copy nuclear loci, in the four studied *Notopterygium* species.

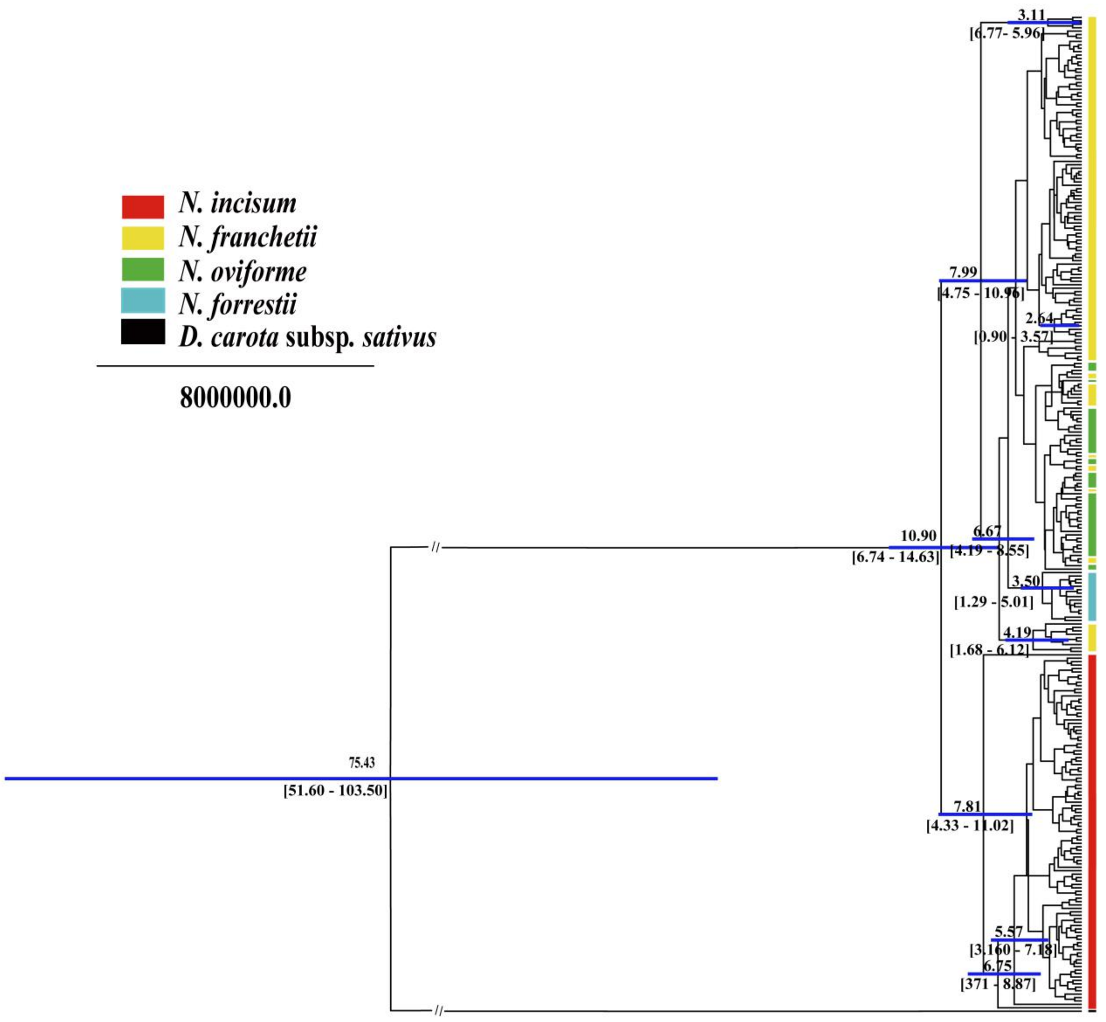

Figure S6 BEAST-derived chronograms of four species based on concatenated 10 single copy nuclear loci, in the four studied *Notopterygium* species.

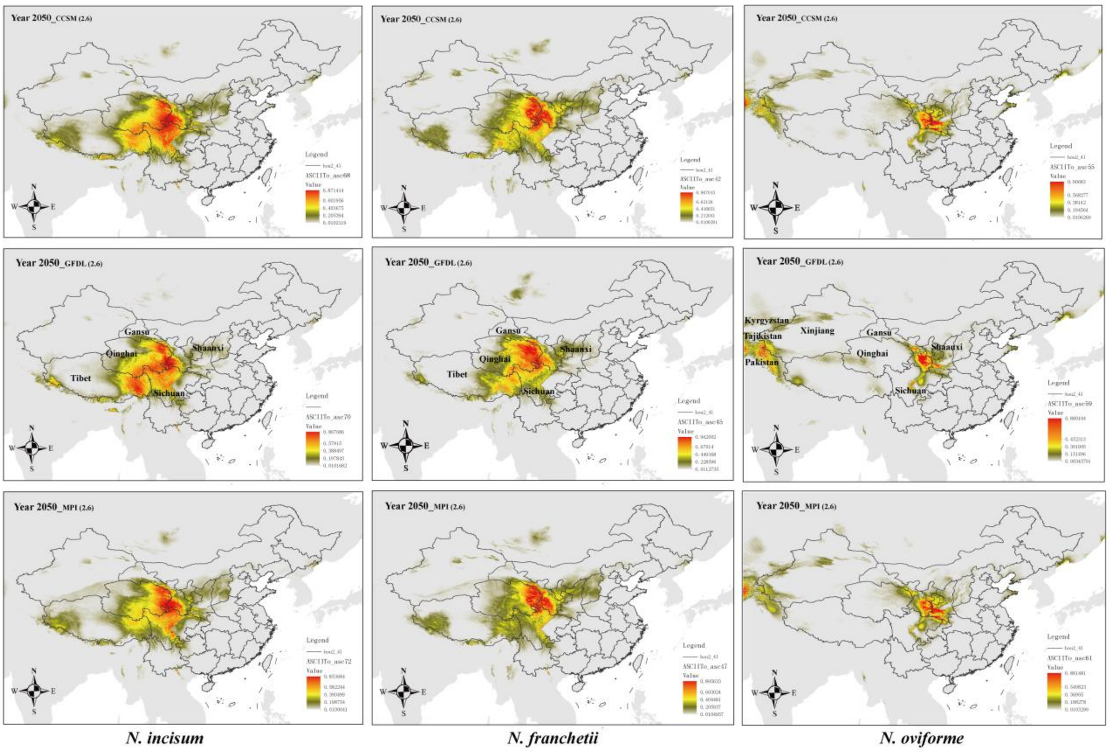

Figure S7 Species distribution modeling results for *N. incisum*, *N. franchetii* and *N. oviforme* for the year 2050 using three models: CCSM, GFDL and MPI based on RCP 2.6. The values of maximum sensitivity plus specificity logistic threshold were 0.1447, 0.1549, and 0.4723 for *N. incisum*, *N. franchetii* and *N. oviforme*, respectively. Pixels below this value should not be considered as suitable for the species.

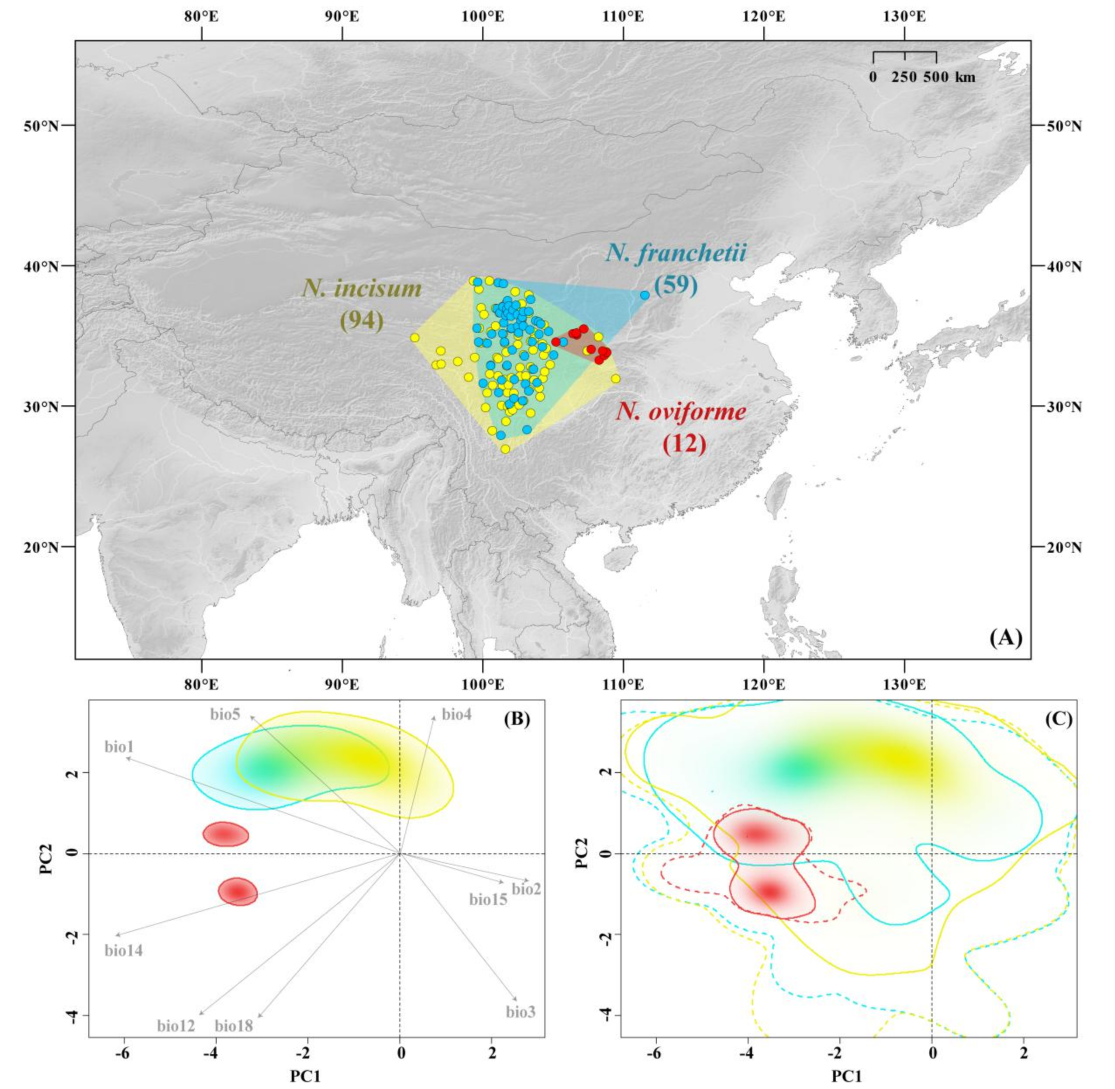

Figure S8 (A) Geographic location of the occurrence records and background areas used for the climatic niche comparison in E-space of three *Notopterygium* species: *N. franchetii* (blue), *N. incisum* (yellow), and *N. oviforme* (red). Number below the species corresponds to the number of occurrences used for each one. **(B)** and **(C)** Global climatic space represented through a PCA-env (explaining PC1 = 52.81% and PC2 = 31.34% of the total climatic variation). In **(B)** the solid lines delimit the 20% of occurrence density of the current occupied niches by the species. Gray arrows outline the direction of variables contribution to the PCA-env. In **(C)** the solid and dashed lines illustrate the 100% of occurrence density and the 100% of available background climates, respectively.
